# CRL3^ARMC5^ ubiquitin ligase and Integrator phosphatase form parallel mechanisms to control early stages of RNA Pol II transcription

**DOI:** 10.1016/j.molcel.2024.11.024

**Published:** 2024-12-11

**Authors:** Roberta Cacioppo, Alexander Gillis, Iván Shlamovitz, Andrew Zeller, Daniela Castiblanco, Alastair Crisp, Benjamin Haworth, Angela Arabiotorre, Pegah Abyaneh, Yu Bao, Julian E. Sale, Scott Berry, Ana Tufegdžić Vidaković

**Affiliations:** 1 Division of Protein and Nucleic Acid Chemistry, MRC Laboratory of Molecular Biology, Cambridge CB2 0QH, UK; 2 EMBL Australia Node in Single Molecule Science, University of New South Wales, Sydney, NSW, Australia; 3 UNSW RNA Institute, University of New South Wales, Sydney, NSW, Australia; 4 Department of Molecular Medicine, School of Biomedical Sciences, University of New South Wales, Sydney, NSW, Australia

## Abstract

Control of RNA polymerase II (RNA Pol II) through ubiquitylation is essential for the DNA-damage response. Here, we reveal a distinct ubiquitylation pathway in human cells, mediated by CRL3^ARMC5^, that targets excessive and defective RNA Pol II molecules at the initial stages of the transcription cycle. Upon ARMC5 loss, RNA Pol II accumulates in the free pool and in the promoter-proximal zone but is not permitted into elongation. We identify Integrator subunit 8 (INTS8) as a gatekeeper preventing the release of excess RNA Pol II molecules into gene bodies. Combined loss of ARMC5 and INTS8 has detrimental effects on cell growth and results in the uncontrolled release of excessive RNA Pol II complexes into early elongation, many of which are transcriptionally incompetent and fail to reach the ends of genes. These findings uncover CRL3^ARMC5^ and Integrator as two distinct pathways acting in parallel to monitor the quantity and quality of transcription complexes before they are licensed into elongation.

## Graphical abstract

**Graphical abstract:**
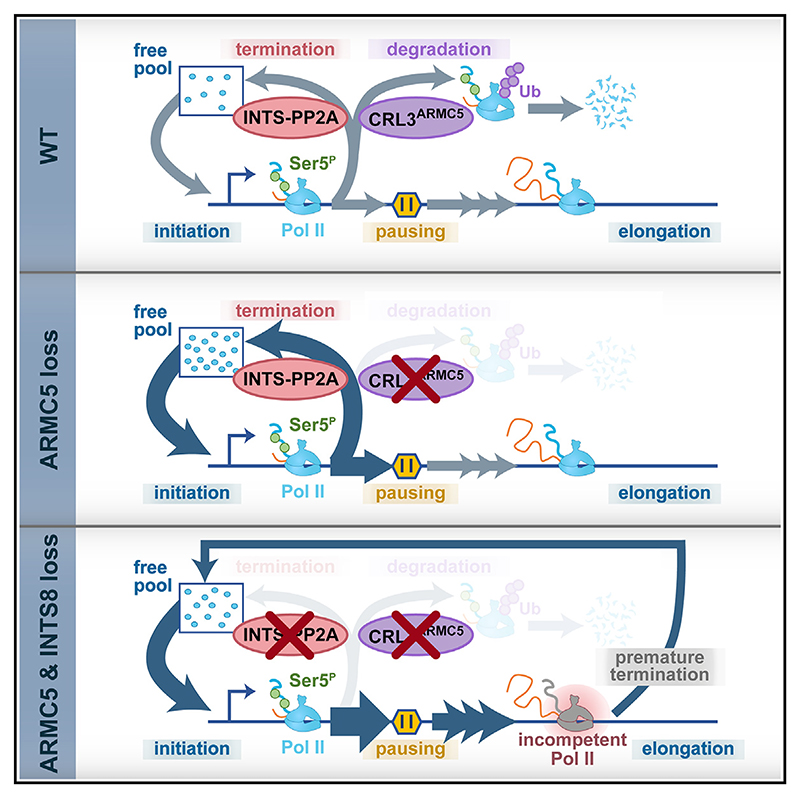

## Introduction

At the heart of the central dogma, RNA polymerase II (RNA Pol II) transcribes all protein-coding genes and thousands of noncoding RNAs in eukaryotes.^1–3^ A human cell possesses around 100,000 RNA Pol II molecules; however, this varies between cells in a population, and it is modified upon perturbation of transcription or RNA degradation.^4–10^ Approximately half the RNA Pol II molecules in a cell are engaged on chromatin, with the remainder either interacting with chromatin transiently or freely diffusing in the nucleoplasm.^8,11–13^ The factors and mechanisms responsible for controlling RNA Pol II abundance remain largely unknown.^14^

In a single transcription cycle, RNA Pol II passes through multiple stages, each providing an opportunity to regulate gene expression.^2,3,15,16^ Transcription factors stabilize RNA Pol II at transcription start sites (TSSs) where it forms a complex with pre-initiation proteins, and then transcription initiates.^17^ At many metazoan genes, RNA Pol II does not immediately proceed to elongation, typically accumulating 20–60 bp downstream of the TSS^15,18,19^ in a region called the promoter-proximal zone.

This zone constitutes a major control point for transcription, where RNA Pol II can be held in a paused state by 5,6-dichloro-benzimidazole 1-β-D-ribofuranoside (DRB)-sensitivity inducing factor (DSIF) and negative elongation factor (NELF), proceed to elongation, or terminate.^15,20,21^ Release into elongation is mediated by cyclin-dependent kinase 9 (CDK9), which phosphorylates both RNA Pol II and DSIF-NELF.^15,22–24^

The transition between transcription initiation and elongation is highly inefficient—RNA Pol II often terminates within the promoter-proximal zone, rather than proceeding to create a full-length transcript.^13,21^ Recent estimates of this ‘premature’ termination are as high as 80% of initiation events in human cells.^8,21^ The possible functions of termination in the early stages of transcription could be to ensure that transcription complexes are competent for elongation, to provide additional opportunities for gene regulation, or to maintain specific chromatin states at promoters.

A key contributor to premature termination is the Integrator complex, which is composed of endonuclease and phosphatase modules that independently impact transcription cycle dynamics.^25–34^ Integrator endonuclease cleaves RNA and terminates transcription within 3 kb downstream of the TSS, proposed to target incorrectly assembled RNA Pol II complexes incompetent for elongation.^25,30,32,33^ Conversely, Integrator phosphatase prevents release of RNA Pol II from pausing, by dephosphorylating both RNA Pol II and DSIF, thus counter-acting CDK9 activity.^27,29,34^

While phosphorylation of RNA Pol II regulates virtually all stages of transcription,^35–38^ it is poorly understood if or how other RNA Pol II post-translational modifications (PTMs), like ubiquitin, impact the transcription cycle. When elongating RNA Pol II stalls on obstacles, it becomes ubiquitylated at lysine 1268 (K1268) on its catalytic subunit, RPB1,^5,39^ and this modification is essential for cells to survive DNA damage.^5^ RPB1 K1268 on elongation-stalled RNA Pol II is targeted by at least two different E3 ubiquitin ligases, Cockayne Syndrome A (CSA) and a yet unknown ligase, leading to either stabilization of the transcription-coupled nucleotide excision repair complex^39,40^ or to RPB1 degradation as the last-resort pathway,^5,41,42^ respectively. Here, we identify a distinct ubiquitin-mediated RNA Pol II control mechanism, whereby Cullin-RING E3 ubiquitin ligase CUL3-ARMC5 (CRL3^ARMC5^) ubiquitylates RNA Pol II to regulate its levels in the free pool and in the promoter-proximal zone, surveying excess and defective RNA Pol II molecules at early stages of transcription. Through a synthetic lethality screen, we identify Integrator phosphatase module subunit 8 (INTS8) as a gatekeeper that compensates for ARMC5 loss by not allowing this excess RNA Pol II accumulated in the promoter-proximal zone to proceed into elongation. Combined loss of ARMC5 and INTS8 unleashes uncontrolled release of RNA Pol II into early elongation; yet, these transcription complexes are not fully competent for elongation and fail to reach gene ends. Interestingly, a specific class of short, TATA-box-containing genes utilize ARMC5-INTS8 mechanism to attenuate their expression. These findings reveal a parallel function of CRL3^ARMC5^ and Integrator in monitoring the quantity and quality of RNA Pol II complexes before they are licensed into elongation.

## Results

### Distinct forms of ubiquitylated RNA Pol II in the transcription cycle

**Figure 1:**
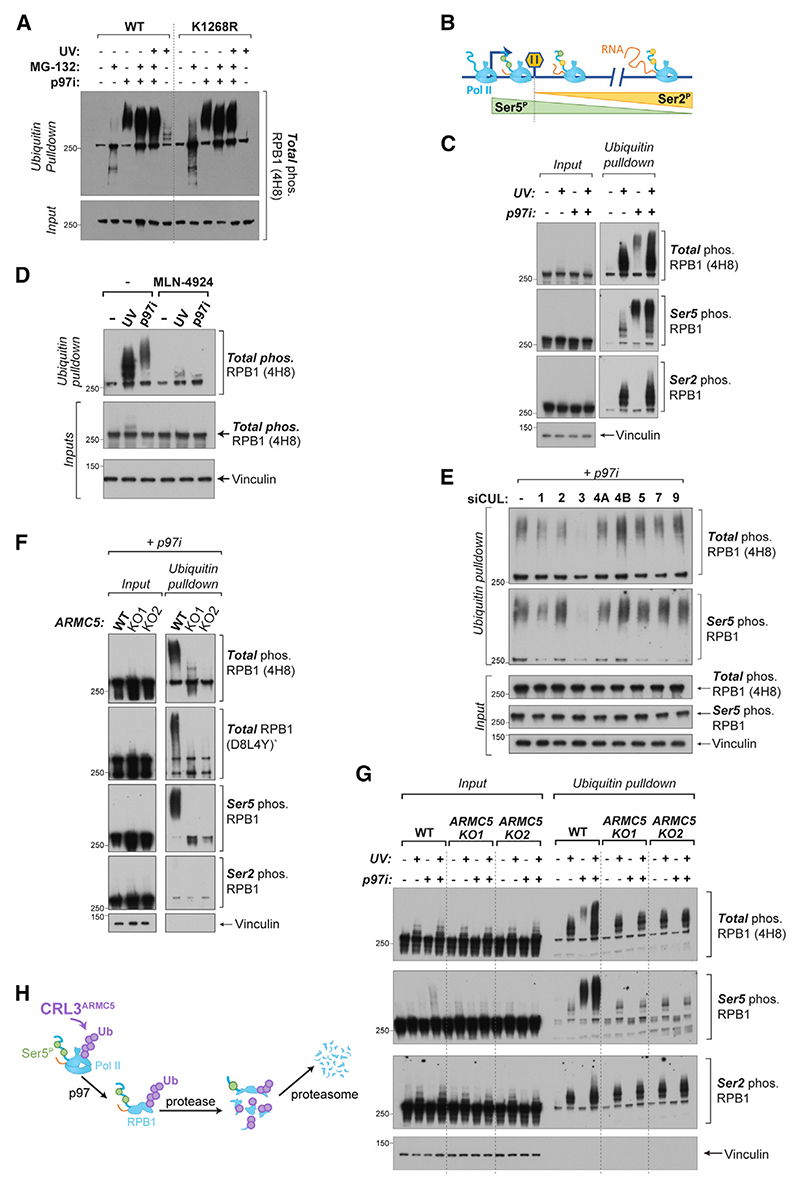
Distinct forms of ubiquitylated RNA Pol II in the transcription cycle (A) Ubiquitin pull-down and western blot in cells expressing wild-type (WT) or K1268R-mutated RPB1 (45 min post-UV, 20 J/m^2^; MG-132, 5 μM for 2 h; p97i = CB-5083, 10 μM for 1 h). (B) Schematic representing the phosphorylation states of RNA Pol II CTD during transcription. (C) As in (A), in WT cells and with CB-5083 (10 μM for 30 min). (D) As in (C), with MLN-4924 treatment (10 μM, pre-treated for 1 h). (E) Ubiquitin pull-down and western blot, siRNA-transfected WT cells (CB-5083, 10 μM for 15 min). (F and G) As in (C), in WT and *ARMC5* KO cells treated with CB-5083 (p97i) alone (F) or in combination with UV (20 J/m^2^, 45 min) (G). (H) A sketch summarizing the CRL3^ARMC5^-mediated RPB1 destruction mechanism. See also Figure S1.

Ubiquitylation of RPB1 K1268 is the only RNA Pol II ubiquitylation event with a clearly ascribed function.^5,39,43,44^ To investigate RNA Pol II ubiquitylation more broadly, we inhibited cellular pathways that process ubiquitylated proteins: the proteasome, which degrades ubiquitylated proteins; and p97/VCP, which unfolds and segregates ubiquitylated substrates from macromolecular complexes to channel them to the proteasome for degradation or allow their recycling.^45^ To facilitate accumulation of normally short-lived ubiquitylated protein species, we chemically inhibited these pathways and analyzed RPB1 ubiquitylation by ubiquitin pull-down and western blot^5,46^ in human HEK293 cells. Using antibodies against RPB1 phosphorylated on its C-terminal domain (CTD) revealed a substantial amount of ubiquitylated RPB1 (Figure 1A). Importantly, this ubiquitylation was not abolished by K1268R mutation of RPB1, which blocks UV-induced RPB1 ubiquitylation^5,39^ (Figure 1A). This therefore represents a specific form of ubiquitylated RNA Pol II, distinct from elongation-stalled RNA Pol II, with a different ubiquitin recipient site or sites. Inhibition of the proteasome by MG132 led to accumulation of ubiquitylated RPB1 fragments, which were abolished when p97 was co-inhibited using CB-5083 (p97i) (Figure 1A). This suggests that this particular form of RPB1 ubiquitylation normally leads to p97-mediated extraction of ubiquitylated RPB1 from the RNA Pol II complex, fragmentation by an unknown cellular protease, and further digestion of fragments by the proteasome.

Phosphorylation of the CTD of RPB1 serves as a marker of different stages of transcription.^35,36,47^ When RNA Pol II initiates transcription, the CTD becomes phosphorylated on Ser5 residues (Ser5^P^). Only upon release into elongation does Ser2 become phosphorylated (Ser2^P^), while Ser5 is progressively dephosphorylated^36–38,47^ (Figure 1B). Elongation-stalled ubiquitylated RNA Pol II was predominantly phosphorylated at Ser2, as expected (Figure 1C, lane 2). Conversely, the distinct form of ubiquitylated RNA Pol II stabilized by p97 inhibition contained Ser5^P^ but no Ser2^P^, indicating that it does not arise from elongating RNA Pol II but rather from earlier stages in the transcription cycle (Figures 1B and 1C, lane 3). Together, these data reveal the existence of an RPB1 ubiquitylation pathway that targets non-elongating RPB1 at a residue distinct from K1268.

Phosphorylation of RPB1 at Ser5 of the CTD is a hallmark of RNA Pol II in the promoter-proximal zone.^35–38,47,48^ Controlling RNA Pol II levels here could provide an immense opportunity for transcriptional regulation. To test if and how RNA Pol II ubiquitylation contributes to this, we set out to identify the E3 ubiquitin ligase responsible, using targeted screening approaches. Inhibition of Cullin-RING family of E3 ubiquitin ligases (CRLs) using MLN-4924 completely abolished the accumulation of poly-ubiquitylated RPB1 following p97 inhibition (Figure 1D), showing that the E3 ligase responsible must belong to this family of enzymes.

CRLs are modular enzymes that rely on an adapter and a substrate receptor to determine substrate specificity.^49^ Eight distinct Cullin proteins can assemble hundreds of different E3 ligases thanks to the diversity of substrate receptors.^49^ To abolish individual branches of cellular Cullin-RING ubiquitylation, we depleted individual Cullins using small interfering RNA (siRNA) and analyzed RPB1 ubiquitylation (Figures 1E and S1A). This showed that Ser5^P^-modified ubiquitylated RPB1 can be detected even without p97 inhibition and that it largely depends on a Cullin 3 (CUL3)-based E3 ligase (Figures 1E and S1A). This agrees with previous work implicating CUL3 and p97 in RPB1 degradation upon depletion of DSIF-subunit SPT5.^7^

The CUL3-specific adapter and substrate receptor ARMC5 has recently been shown to directly interact with RNA Pol II and to mediate its ubiquitylation in cells and in animals,^50,51^ but the mechanism, function, and the consequence for the transcription process remain unknown. To ascertain whether the RPB1 ubiquitylation we observe on Ser5^P^ RNA Pol II is CRL3^ARMC5^ dependent, we generated *ARMC5* knockout (KO) HEK293 cell lines (Figures S1B and S1C) and analyzed RNA Pol II ubiquitylation. Loss of ARMC5 completely abolished ubiquitylation of Ser5-phosphorylated RPB1 (Figure 1F). Importantly, we found that CRL3^ARMC5^ is specific for RNA Pol II originating from the promoter-proximal zone: it does not ubiquitylate elongation-stalled RNA Pol II (Figure 1G), nor is it necessary for its degradation (Figure S1D). We conclude that ARMC5-dependent RPB1 ubiquitylation therefore targets different RNA Pol II species, at a different residue on RPB1, and performs a distinct regulatory role from the previously characterized pathways targeting elongation-stalled RNA Pol II (Figure 1H).

### ARMC5-dependent RNA Pol II degradation is a major turnover pathway during homeostasis

**Figure 2:**
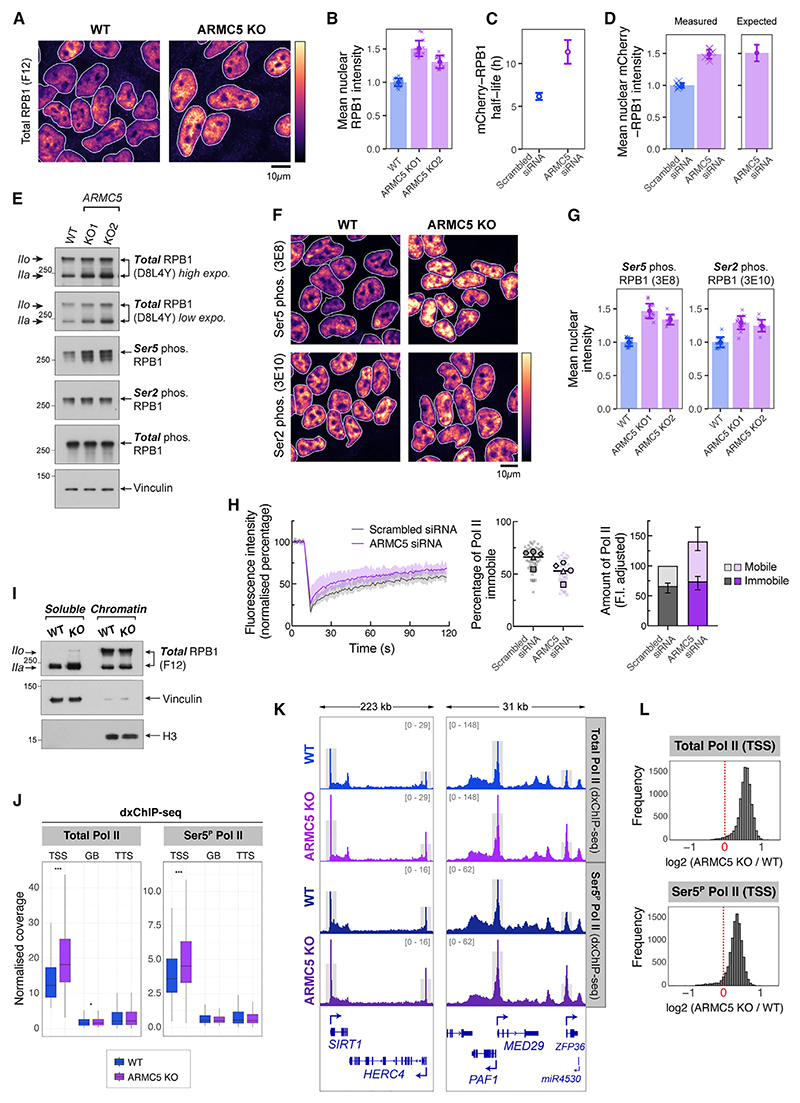
ARMC5 controls the levels of free and promoter-proximal RNA Pol II (A) Example images of total RPB1 immunofluorescence. Maximum-intensity projections shown with overlaid nuclear segmentations. *ARMC5* KO 2 is shown. (B) Quantification of mean nuclear RPB1 intensity from (A). 12–16 replicate wells across two experiments (1,500–3,500 cells/replicate). Error bars show SD. (C) Mean mCherry-RPB1 half-life calculated from bleach-chase experiments. Error bars show 95% CI for the mean (four replicates, 6,500–16,000 cells/replicate). (D) Mean nuclear fluorescence intensity of mCherry-RPB1 in live cells compared with that expected from measured half-life change. Error bars show 95% CI for the mean. (E) Western blot of RPB1 in whole-cell lysates of WT and *ARMC5* KO HEK293 cells. (F and G) As in (A) and (B), respectively, but for the Ser5^P^ and Ser2^P^ forms of RPB1. Error bars show SD. (H) FRAP of mCherry-RPB1 cells following ARMC5 knockdown (50 cells per condition across 5 experiments). Immobile RNA Pol II percentage estimated per cell (small points) and per experiment (large points). FI-adjusted amount of RNA Pol II shows mean with range across experiments. (I) Chromatin fractionation and western blot in WT and *ARMC5* KO, HEK293 cells. (J) dxChIP-seq, boxplots showing the abundance of total (D8L4Y) and Ser5P RNA Pol II in different genomic bins: TSS, transcription start site; GB, gene body; TTS, transcription termination site. Asterisks denote significance determined by Wilcoxon rank-sum test. (K) dxChIP-seq, individual gene examples. Gray boxes indicate RNA Pol II TSS-proximal signal in WT cells. (L) dxChIP-seq, distribution of differences in RNA Pol II abundance at the TSS-proximal region between *ARMC5* KO and WT cells. x axis: log2 ratio (*ARMC5* KO/WT); y axis: number of genes. Genes with coverage >53 over background are analyzed. See also Figures S2 and S3 and Video S1.

Primary bilateral macronodular adrenal hyperplasia (PBMAH)^52^ patients carrying *ARMC5* mutations over-accumulate RPB1 in adrenal glands and other organs.^50^ Similarly, *Armc5* KO mice show elevated RPB1 levels across the animal and in cultured embryonic fibroblasts,^50^ suggesting a pervasive, conserved role for ARMC5 in controlling RNA Pol II abundance. In agreement, *ARMC5* KO cells as well as HCT116 cells transfected with siRNAs targeting *ARMC5* showed strongly elevated levels of nuclear RPB1 by immunofluorescence (Figures 2A, 2B, S2A, and S2B). To test whether this increase in RPB1 levels is driven by impaired RPB1 turnover, we performed bleach-chase assays^53^ to measure the half-life of RPB1 protein in HCT116 cells where both copies of RPB1 are N-terminally tagged with mCherry (Figures S2C–S2G; Video S1). Upon *ARMC5* depletion, mean mCherry-RPB1 half-life almost doubled from 6.2 to 11.4 h during normal cellular growth (Figures 2C and S2H–S2J). This increased RPB1 stability is sufficient to explain the increase in RPB1 levels (Figure 2D), indicating that loss of ARMC5 changes only the rate of RPB1 degradation, without affecting the rate of RPB1 synthesis. After *ARMC5* depletion, the remaining active RPB1 degradation rate approached the rate of dilution due to cell growth (Figure S2J), demonstrating that ARMC5 is essential for a major RPB1 turnover pathway under homeostatic cell growth conditions. The relatively short protein half-life of RPB1 in unperturbed cells is also consistent with our earlier observations that only a short pulse (30 min–1 h) of p97 inhibition results in accumulation of a substantial amount of ubiquitylated RPB1 (Figures 1A and 1B).

### ARMC5 controls the levels of free and promoter-proximal RNA Pol II

We next investigated which part of the transcription cycle may be affected by ARMC5 loss. On western blots probed with CTD-independent antibodies, RPB1 manifests two distinct bands. ARMC5 depletion predominantly causes an increase in abundance of the lower RPB1 band, IIa (Figures 2E and S3A), which corresponds to largely unphosphorylated or lowly Ser5-phosphorylated RPB1.^54^ Additionally, *ARMC5* KO cells displayed a substantial increase in RPB1 Ser5^P^ signal, while Ser2^P^ was less affected (Figures 2E–2G and S3A). Together with our earlier observation that ARMC5-mediated ubiquitylation is found on RNA Pol II modified with Ser5^P^ but not Ser2^P^ (Figures 1B–1G), this further indicates that ARMC5 regulates RNA Pol II at the initial steps of transcription.

To investigate RNA Pol II dynamics in living cells, we performed fluorescence recovery after photobleaching (FRAP) assays using HCT116 mCherry-RPB1 cells. Two primary populations of RPB1 were detected, one with high mobility that displayed fast recovery after bleaching and a second component showing very slow recovery and therefore having low mobility or being immobilized (Figures S3B–S3I). We assume that the high-mobility state comprises freely diffusing RNA Pol II molecules, as well as those that are not stably bound to chromatin. The stably bound fraction comprises elongating and stably paused RNA Pol II. Fitting two-component recovery curves to FRAP data (Figure S3G) indicated that the half-life of the mobile and stably bound components was 3 and 139 s, respectively. In unperturbed cells, we estimate that 60% of RPB1 is stably bound, in agreement with similar assays of RPB1 performed previously in other systems^8,11,12^ (Figure 2H). Upon *ARMC5* depletion, the fraction of mobile RPB1 was greatly increased (Figure 2H), indicating that a large proportion of RPB1 is not stably bound to chromatin. After correcting for the increase in overall RPB1 levels, the absolute amounts of stably bound RPB1 remained unchanged. In agreement with FRAP, chromatin fractionation followed by western blot also indicated that excess RPB1, accumulating in *ARMC5* KO cells, was found mostly in the soluble fraction, rather than purifying biochemically with chromatin (Figure 2I). Excess RPB1 that accumulates upon ARMC5 removal is therefore predominantly found in the mobile fraction—either in the free pool or in rapid cycles of initiation and termination, rather than being stably associated with chromatin.

To further investigate this, we mapped RNA Pol II occupancy across the genome using double-crosslinking chromatin immunoprecipitation and sequencing (dxChIP-seq). In *ARMC5* KO, both total RNA Pol II and Ser5^P^ RNA Pol II specifically accumulated close to the TSSs—in the promoter-proximal zone, but not further in gene bodies or at transcription termination sites (TTSs) (Figures 2J, 2K, and S3J), indicating that excess RNA Pol II at the promoter-proximal region is somehow prevented from entering elongation. This is consistent with the relatively constant levels of immobile RNA Pol II observed via FRAP. The effect of *ARMC5* KO was apparent on almost all RNA Pol II-transcribed genes (Figure 2L), suggesting that ARMC5 affects promoter-proximal RNA Pol II globally.

### ARMC5 targets perturbed early transcription complexes

**Figure 3:**
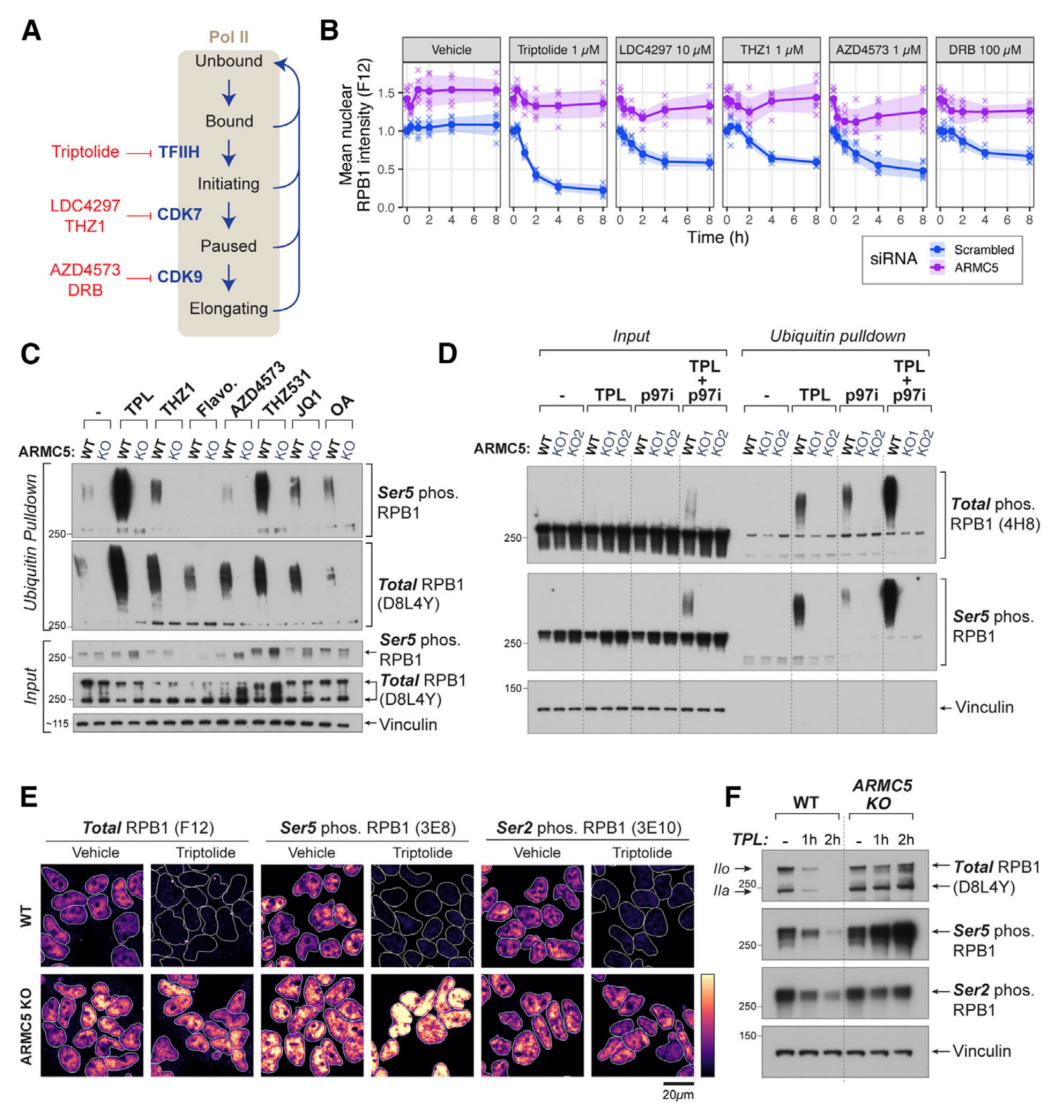
ARMC5 targets perturbed early transcription complexes (A) Schematic of the transcription cycle indicating steps targeted by the inhibitors used in immunofluorescence experiments. (B) Mean nuclear intensity of total RPB1, detected via immunofluorescence with F12 N-terminal antibody, HCT116 cells. Mean ± SD of six replicates from three experiments. (C) Ubiquitin pull-down and western blot (TPL, triptolide: 300 nM for 1 h; THZ1: 250 nM for 1 h; flavopiridol: 5 μM for 15 min; AZD4573: 500 nM for 1 h; THZ531: 500 nM for 6 h; JQ1: 5 μM for 3 h; OA, okadaic acid: 500 nM for 1 h) all in combination with CB-5083 p97i (10 μM for 30 min). (D) As in (C), with TPL and CB-5083 (p97i) alone or in combination. (E) Example images of total, Ser5^P^, and Ser2^P^ RPB1, detected via immunofluorescence (HEK293 cells) treated with 300 nM TPL or 0.4% DMSO vehicle for 4 h. (F) Western blot detecting different forms of RPB1 in whole-cell lysates (HEK293) treated with 300 nM TPL. See also Figure S4.

Inducing defects in RNA Pol II initiation, pausing, and elongation can lead to RPB1 degradation.^5–8,10,41,46,55–57^ To test if ARMC5 is involved in this perturbation-induced loss of RNA Pol II, we monitored RPB1 levels in HCT116 cells transfected with *ARMC5* siRNAs, upon treatment with a variety of transcription inhibitors. Inhibitors were chosen to target key steps in the promoter-proximal zone: triptolide (inhibits TFIIH subunit XPB, a translocase that facilitates promoter melting during transcription initiation)^56,57^; LDC4297 and THZ1 (inhibit CDK7^58,59^ responsible for Ser5 phosphorylation, release from the enhancer-bound Mediator complex and recruitment of SPT5); and DRB and AZD4573 (inhibit CDK9^24,60^ that phosphorylates RNA Pol II CTD Ser2, SPT5, and NELF, releasing RNA Pol II from the promoter-proximal zone into elongation) (Figure 3A). Strikingly, perturbation-induced loss of RPB1 was almost completely blocked in *ARMC5*-depleted cells (Figure 3B), demonstrating that ARMC5 is essential for degradation of RNA Pol II in perturbed early transcription complexes. Similar results were obtained in HEK293 *ARMC5* KO cells, where we measured both total RPB1 abundance as well as RPB1 CTD phosphorylation levels (Figure S4A).

Ubiquitin pull-down in the presence of p97 inhibitor confirmed the increase in ubiquitylated RPB1 upon chemical perturbation of transcription, which was fully dependent on ARMC5 in all cases tested (Figure 3C), using an expanded inhibitor panel (JQ1 that inhibits BET family of bromodomain proteins that stimulate CDK9 and pause-release^61^; okadaic acid that inhibits PP2A,^62^ responsible for dephosphorylating RNA Pol II in the promoter-proximal zone^27,29,34^; and THZ531 that inhibits CDK12/13,^63^ responsible for phosphorylating RNA Pol II at Ser2 during elongation^64^) (Figure S4B). Together, these results reveal that a broad range of inhibitors induce ARMC5-dependent RPB1 ubiquitylation and degradation.

In both wild-type (WT) and *ARMC5* KO cells, effects were most dramatic with triptolide, which triggered ARMC5-mediated RPB1 ubiquitylation to the greatest extent (Figure 3C) and caused an almost complete loss of RNA Pol II in WT cells but no loss in *ARMC5*-depleted cells (Figures 3B and S4A). Upon short (30 min) triptolide treatment, ARMC5-dependent ubiquitylation can be detected by western blot in the presence of p97 inhibitor, even without enriching ubiquitylated proteins by pull-down (Figure 3D, input). These findings demonstrate that ARMC5 is capable of ubiquitylating most cellular RNA Pol II molecules within hours when the transcription cycle is perturbed. Importantly, triptolide-mediated RPB1 degradation still occurs in RPB1 K1268R mutant, again highlighting that the last-resort pathway^5,41,42^ and ARMC5 regulate distinct forms of RNA Pol II (Figure S4C).

Triptolide-mediated inhibition of XPB has been assumed to completely block transcription initiation.^65^ Surprisingly, we observed that following triptolide exposure, *ARMC5* KO cells show not only increased levels of Ser5^P^ RPB1 but also persistence of Ser2^P^, as observed both by western blots and immuno-fluorescence (Figures 3E, 3F, and S4A). This raises an intriguing possibility that transcription elongation may be possible in the presence of triptolide when ARMC5 is absent.

### Loss of ARMC5 confers partial resistance to triptolide

**Figure 4:**
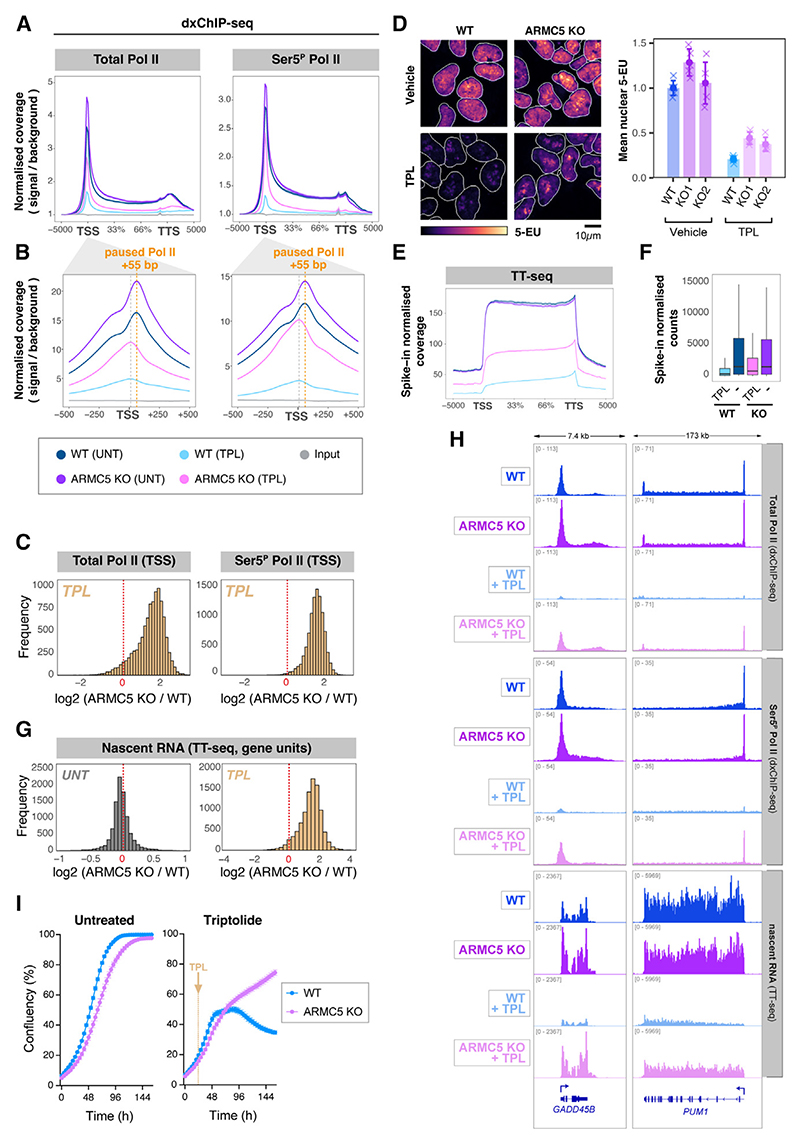
ARMC5 loss confers partial resistance to XPB inhibition by triptolide (A) dxChIP-seq, metagene profiles of RNA Pol II occupancy (TPL, 300 nM for 2 h). Legend shown in (B). (B) Zoom-in of (A) around the TSS. x axis: relative scale for (A) (TSS and TTS are indicated) and absolute scale for (B); y axis: read counts normalized to background. (C) dxChIP-seq, distribution of differences in RNA Pol II abundance at the TSS. x axis, log2 ratio (*ARMC5* KO/WT); y axis, number of genes. Genes with coverage >53 over background are analyzed. (D) Example images (nuclear segmentations overlaid in white) and relative mean nuclear 5EU intensity for HEK293 cells pulse labeled for 30 min. TPL, 300 nM for 2 h. Mean ± SD of 5–6 replicates from 3 experiments (500–2,000 cells/replicate). (E) Spike-in normalized metagene TT_chem_-seq profiles showing nascent RNA distribution across gene units (TPL, 300 nM for 2 h). (F) Boxplots showing total spike-in normalized TT_chem_-seq read counts at expressed genes (>10 normalized reads). (G) TT_chem_-seq, distribution of differences in nascent RNA abundance on individual genes, without (left) and with (right) TPL. x axis: log2 ratio (*ARMC5* KO/WT); y axis: number of genes. (H) Individual gene examples from dxChIP-seq (top, middle) and TT_chem_-seq (bottom) experiments. Note that *GADD45B* is 1 of the 44 genes induced by *ARMC5* KO in untreated condition; this is explored further in Figure 7. (I) Cell growth assay in WT and *ARMC5* KO cells, without (left) and with (right) TPL (5 nM). Representative of biological triplicate experiment is shown; data re represented as mean ± standard error of imaging. See also Figure S5.

To test whether RNA Pol II can initiate and elongate in the presence of triptolide in *ARMC5* KO cells, we used dxChIP-seq to map total and Ser5^P^ RNA Pol II occupancy genome-wide. A significant amount of RNA Pol II remained bound to genes upon triptolide exposure in *ARMC5* KO cells (Figures 4A and 4B). The effect of ARMC5 loss is global, with the vast majority of genes retaining TSS-proximal RNA Pol II upon triptolide treatment (Figure 4C).

We next estimated nascent RNA production on a global scale using short pulses of RNA labeling with nucleoside analog 5-ethynyl uridine (5EU).^66^ We did not detect a reproducible change in 5EU incorporation in untreated *ARMC5*-depleted cells, compared with the WT (Figures 4D and S5A). However, *ARMC5* KO cells did retain an increased fraction of their RNA synthesis activity upon treatment with triptolide (Figure 4D), suggesting that loss of ARMC5 allows RNA Pol II (which would normally be degraded) to bypass triptolide-inhibited XPB and synthesize RNA. This was confirmed by analyzing nascent RNA synthesis genome-wide, using transient transcriptome sequencing with chemical fragmentation of RNA (TT_chem_-seq)^67^ (Figures 4E, 4F, and S5B), which showed this effect was global, affecting the vast majority of genes (Figures 4G, 4H, S5B, and S5C).

Triptolide is normally toxic to cells.^56^ Surprisingly, a low dose (5 nM) of triptolide killed WT cells but not *ARMC5* KO cells (Figure 4I), revealing that at least some of the inhibitory and toxic effects of triptolide are due to the ARMC5-dependent RNA Pol II degradation. Together, these data demonstrate that RNA Pol II can (partially) bypass inhibited XPB translocase to initiate and elongate transcripts if it is not first targeted for degradation by ARMC5.

### ARMC5 loss causes accumulation of evicted, phosphorylated RNA Pol II off-chromatin

**Figure 5:**
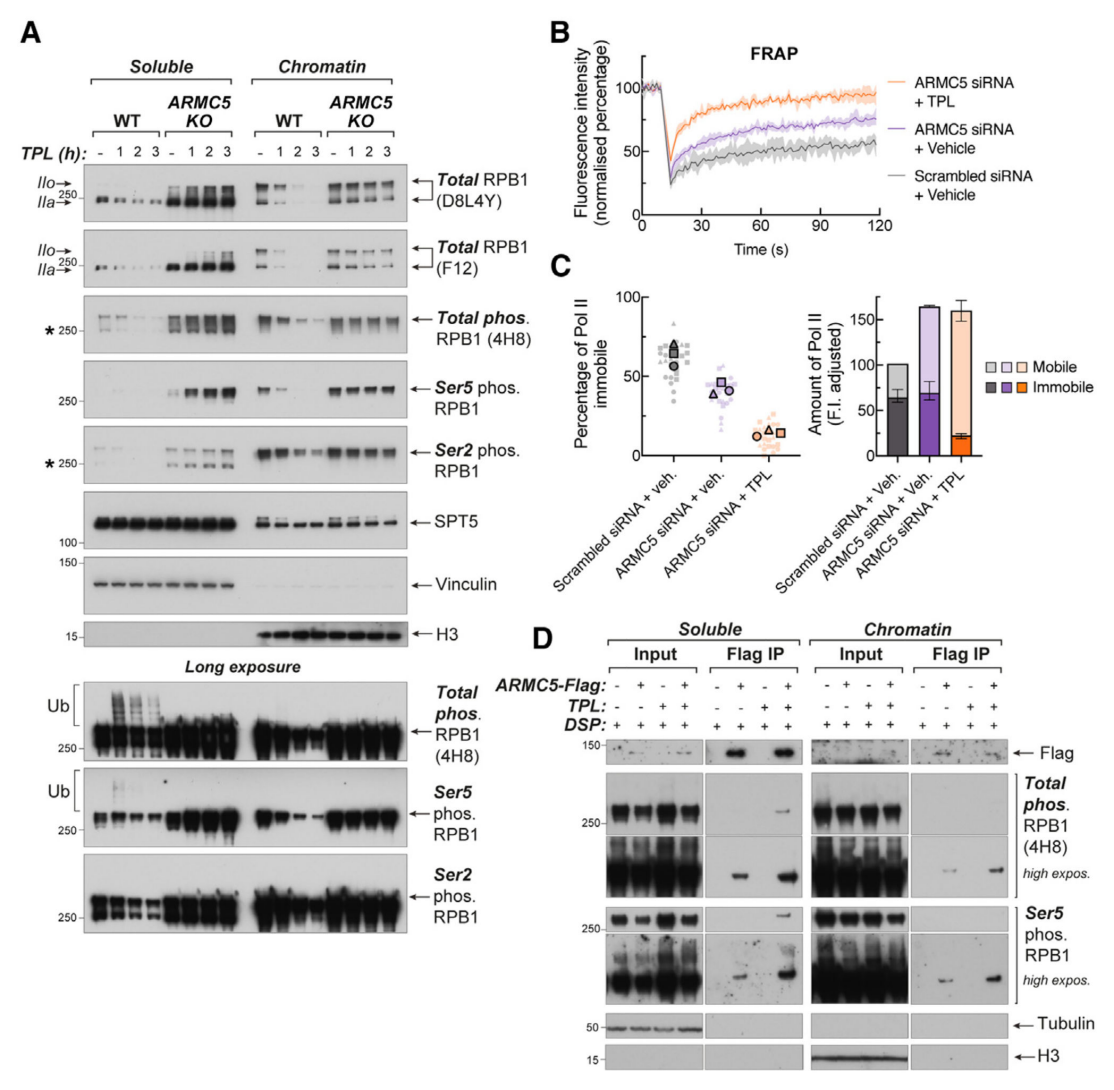
Evicted, phosphorylated RNA Pol II accumulates off-chromatin in the absence of ARMC5 (A) Chromatin fractionation and western blot (TPL, 300 nM). Asterisks denote partially dephosphorylated RPB1. (B) FRAP of mCherry-RPB1 cells treated with *ARMC5* siRNA or scrambled siRNA control and triptolide or vehicle. Mean with range of three experiments (total of 25–27 cells per condition). (C) Immobile RNA Pol II percentage estimated from FRAP experiments per cell (small points) and per experiment (large points). Total RNA Pol II amount adjusted for FI shown as mean with range of experiments. (D) Chromatin fractionation followed by FLAG-IP and western blot in *ARMC5* KO cells, transfected with an empty vector or an ARMC5-FLAG construct, treated with DSP (20 mM) for 30 min and with vehicle or triptolide (300 nM) for 1 h. See also Figure S6.

While some RNA Pol II molecules bypass inhibited XPB in *ARMC5* KO cells and proceed into elongation, we observed that a significant fraction of phosphorylated RPB1 is progressively lost from chromatin upon triptolide treatment, accumulating in the soluble fraction (Figure 5A). Importantly, phosphorylated RPB1 also accumulates in the soluble fraction even in untreated *ARMC5* KO cells (Figure 5A). Our current understanding is that RNA Pol II can only be phosphorylated on chromatin,^35–38,47,48,54^ which suggests that phosphorylated soluble RPB1 is evicted from chromatin without being dephosphorylated. Moreover, when ARMC5-mediated RPB1 degradation was prevented, treatment with triptolide led to a significant increase in the fraction and absolute amount of mobile RNA Pol II observed by FRAP in live cells—well beyond the (already elevated) levels of mobile RNA Pol II seen in *ARMC5*-depleted HEK293 or HCT116 cells (Figures 5B, 5C, S6A, and S6B). Together, these results show that preventing ARMC5-dependent RNA Pol II degradation causes accumulation of evicted, phosphorylated RNA Pol II off-chromatin, which is further exacerbated when initiation is perturbed with triptolide.

On long exposures of western blots, ARMC5-dependent ubiquitylated RPB1 traces were predominantly observed in the soluble fraction (Figure 5A, bottom panels). To determine if ARMC5 interacts with chromatin, we performed fractionation and ARMC5 immunoprecipitation experiments, which revealed that over-expressed ARMC5 is predominantly found in the soluble fraction (in agreement with its previously reported diffuse nuclear and cytoplasmic localization^50^) (Figure S6C). However, when crosslinker dithiobis (succinimidyl propionate) (DSP) was used,^68^ ARMC5 could also be detected in the chromatin fraction, along with the interaction between ARMC5 and RPB1 (Figure 5D). When no crosslinker was used, the RPB1-ARMC5 interaction was observed only in the soluble fraction (Figure S6C). This indicates that ARMC5 is found predominantly in the soluble fraction, where it interacts with RNA Pol II, but it may also transiently interact with RNA Pol II on chromatin.

Two possibilities can explain these results, and they are not mutually exclusive: (1) ARMC5 may ubiquitylate promoter-proximal RNA Pol II on chromatin with RNA Pol II and ARMC5 then rapidly evicted from the DNA into the soluble fraction; and/or ARMC5 may ubiquitylate RNA Pol II in the free pool, after eviction by another factor. Either scenario is compatible with effects of ARMC5 depletion observed throughout this study.

### Integrator phosphatase module compensates for the loss of ARMC5

Regardless of whether ARMC5 targets RNA Pol II on chromatin or in the free pool, RNA Pol II dxChIP-seq (Figures 2J–2L) and TT_chem_-seq in unperturbed cells (untreated condition in Figures 4E and S5C) reveal that excess RNA Pol II accumulates in the promoter-proximal zones of genes upon ARMC5 loss and not in gene bodies.

**Figure 6:**
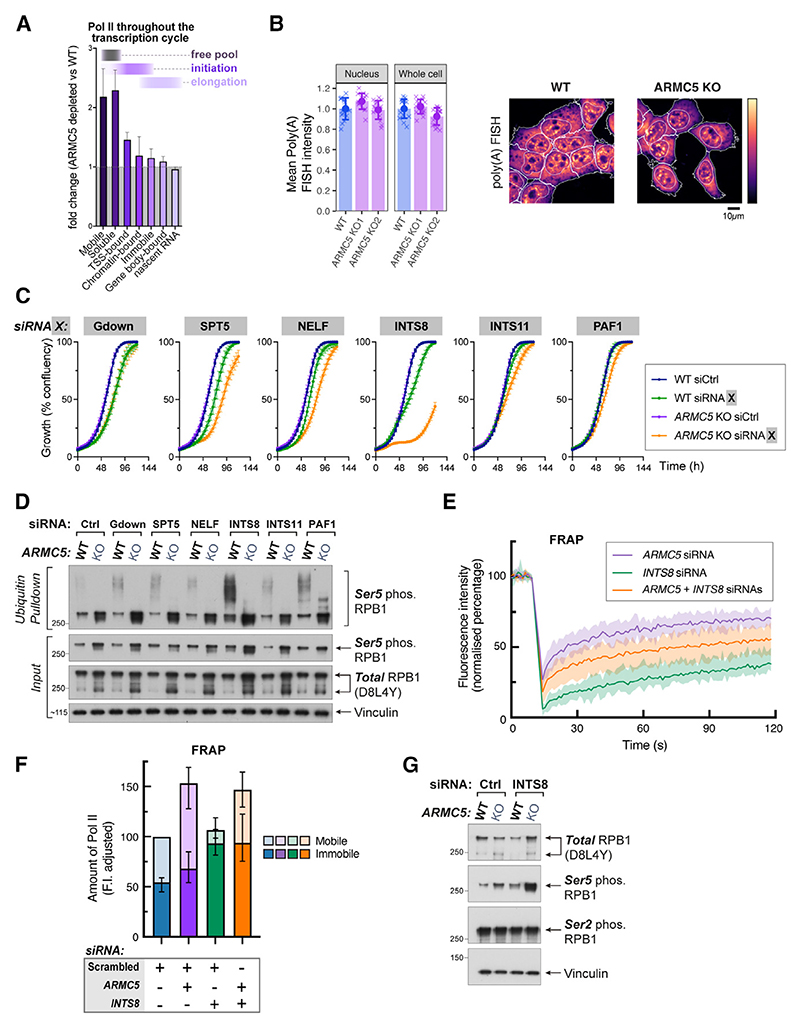
Integrator phosphatase module compensates for the loss of ARMC5 (A) Summary of ARMC5 effects on RNA Pol II at different stages of the transcription cycle—quantification of data obtained throughout the study. ARMC5 ‘depleted’ refers to siRNA-mediated knockdown of *ARMC5* in FRAP and to *ARMC5* KO in all other cases. ‘Mobile’: RNA Pol II fraction in FRAP; ‘soluble’: RNA Pol II in chromatin fractionation; ‘TSS-bound’: RNA Pol II in dxChIP-seq; ‘chromatin-bound’: RNA Pol II in chromatin fractionation; ‘immobile’: RNA Pol II in FRAP; ‘gene body-bound’: RNA Pol II in dxChIP-seq; ‘nascent RNA’: shown in TT_chem_-seq. Details of quantification and statistical analyses are in STAR Methods. (B) Example images and quantification of poly(A) FISH (HEK293 cells). Mean ± SD of 10 replicates across 2 experiments (500–6,000 cells/replicate). (C) Cell growth assays in wild-type and *ARMC5* KO cells, transfected with indicated siRNAs, monitored by Incucyte. Representative of biological triplicates (each with 6 technical replicate wells) is shown; data are shown as mean with standard error of imaging. (D) Ubiquitin pull-down and western blot, the same conditions as in (C). (E) FRAP of mCherry-RPB1 cells following ARMC5 knockdown and INTS8 knockdown alone or in combination. Mean with range of five experiments (total of 50 cells per condition). (F) Total RNA Pol II amount adjusted for FI. Mean with range across experiments. (G) Western blot detecting total RPB1, Ser5^P^, and Ser2^P^, upon *ARMC5* KO and *INTS8* knockdown. See also Figure S7 and Video S2.

To comprehensively quantify the consequences of ARMC5 loss on each stage of the transcription cycle, we used data collected throughout this study (Figure 6A). *ARMC5* depletion most severely affects mobile and free RNA Pol II molecules, moderately affects the levels of promoter-proximal RNA Pol II on chromatin, and does not largely affect gene body RNA Pol II occupancy or transcriptional activity (Figure 6A). Using poly(A) fluorescence *in situ* hybridization (FISH), we also determined that ARMC5 loss does not affect abundance of polyadenylated RNA (Figure 6B). These data suggest that each successive step in the transcription cycle progressively buffers the effect of *ARMC5* depletion: despite excess RNA Pol II in the free pool, only a fraction is recruited and retained in the promoter-proximal zones of genes, and despite increased levels of RNA Pol II in the promoter-proximal zones, only a fraction of those are released into productive elongation. Therefore, control mechanisms acting in the promoter-proximal zone can compensate if excess RNA Pol II is present at the transcription initiation stage, which prevents it from entering gene bodies.

We reasoned that removal of such control mechanisms in combination with ARMC5 loss may compromise the ability of the cell to adjust to increased levels of RNA Pol II, resulting in synthetic lethality. *ARMC5* KO cells are viable with no apparent growth phenotype. We were therefore able to perform a synthetic lethality screen, using siRNAs to target known key regulators of promoter-proximal transcription, in WT and *ARMC5* KO cells. In particular, we depleted the following: Gdown (prevents premature release from the initiation complex),^69^ SPT5 (component of DSIF, keeps RNA Pol II in a stably paused complex; when phosphorylated, it mediates release from pausing and turns into a positive elongation factor),^15,23^ NELF (keeps RNA Pol II stably paused),^22^Integrator phosphatase module subunit INTS8 (prevents excessive pause-release via dephosphorylating RNA Pol II and SPT5),^27,29,34^ Integrator RNA cleavage module sub-unit INTS11 (attenuates transcription via premature termination),^25,30,32–34^ and PAF1 (keeps RNA Pol II stably paused and converts to a positive elongation factor upon pause-release)^70^ (Figure S7A). This revealed that depletion of *INTS8*, but not other factors, has a strong synthetic growth-retardation phenotype with ARMC5 loss (Figure 6C). Importantly, *INTS8* depletion in *ARMC5* KO cells did not obviously increase cell death, allowing these cells to be used in subsequent analyses (Video S2). Notably, depleting any of the promoter-proximal factors triggered some level of ARMC5-mediated RPB1 ubiquitylation, with *INTS8* depletion causing the most pronounced effect (Figure 6D).

Monitoring RNA Pol II dynamics by FRAP revealed that *INTS8* knockdown alone substantially delayed fluorescence recovery (Figure S7B), indicating that the vast majority of RNA Pol II molecules in *INTS8*-depleted cells are stably bound to chromatin (Figure S7C). Similarly, a combined knockdown of *INTS8* and *ARMC5* slowed the FRAP kinetic of mCherry-RPB1 when compared with the *ARMC5*-only knockdown condition (Figure 6E). After correcting for changes in overall RNA Pol II abundance (Figures S7D and S7E), we observed that the effects of *ARMC5* and *INTS8* depletion are largely separable: *ARMC5* depletion results in excessive cellular RNA Pol II, most of which accumulates in the mobile fraction (representing RNA Pol II in the free pool and possibly in rapid cycles of initiation-termination), while *INTS8* depletion causes retention of RNA Pol II molecules in the immobile fraction (likely representing stably paused and elongating RNA Pol II), regardless of the ARMC5 status (Figure 6F). Interestingly, while total RNA Pol II levels depend on ARMC5 but not on INTS8, western blots revealed that Ser5^P^, a mark of promoter-proximal RNA Pol II, depends on both factors. Moreover, these effects are additive (Figures 6D and 6G). Together, these results suggest that ARMC5 and INTS8 regulate different aspects of RNA Pol II dynamic behavior, which somehow converge to regulate levels of Ser5-phosphorylated RNA Pol II.

### Integrator phosphatase and ARMC5 control early transcription complexes through parallel mechanisms

**Figure 7:**
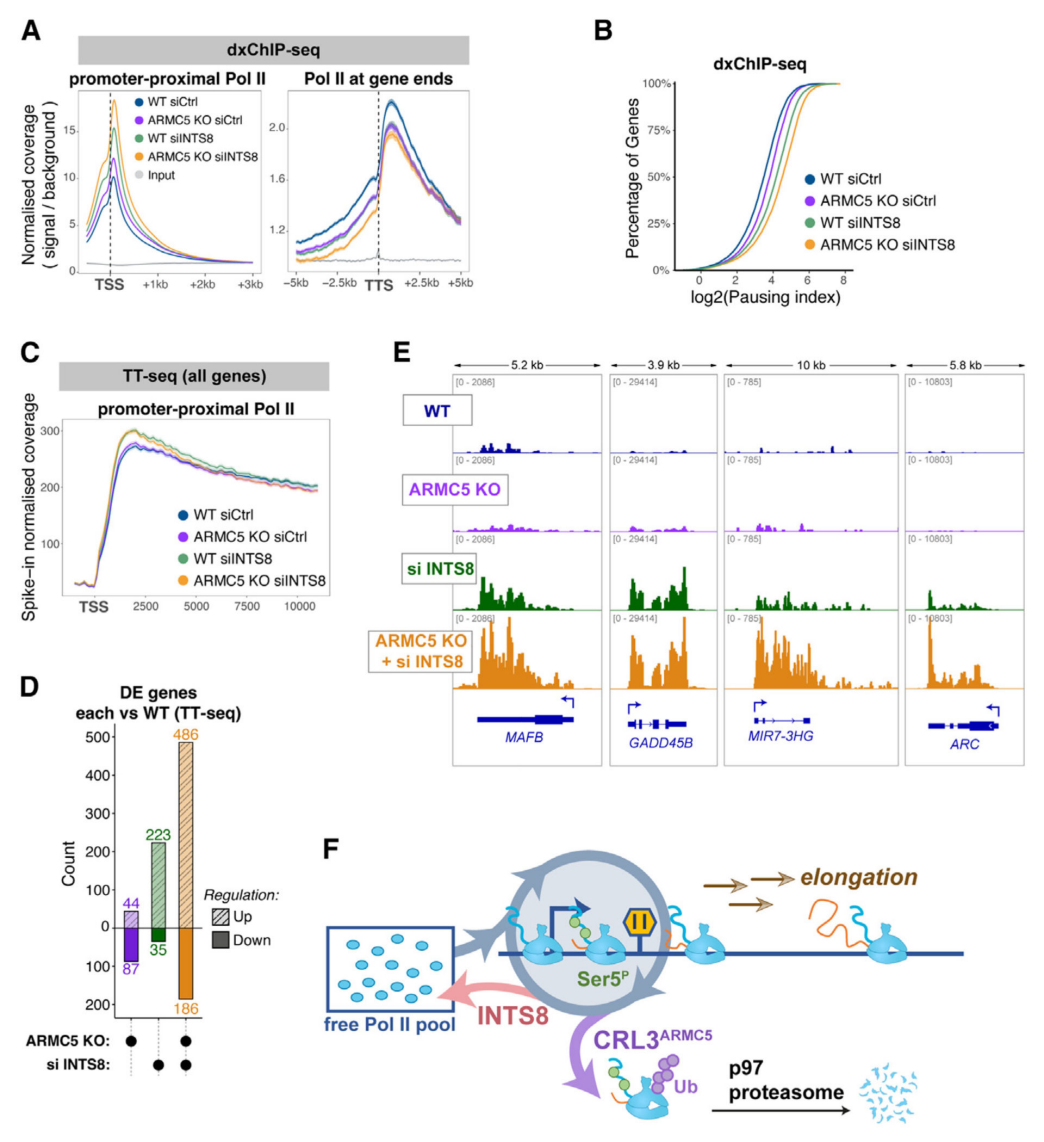
ARMC5 and INTS8 regulate the quantity and quality of early transcription complexes (A) dxChIP-seq, metagene profiles of RNA Pol II occupancy. x axis: TSS and TTS are indicated, absolute scale; y axis: read counts normalized to background. (B) RNA Pol II pausing index in each dxChIP-seq condition. (C) Spike-in normalized metagene TT_chem_-seq profiles showing nascent RNA distribution in the first 10 kb of genes (genes >10 kb were considered). (D) Number of differentially expressed genes (TT_chem_-seq) detected in *ARMC5* KO, knockdown of INTS8 (siINTS8) or a combination of *ARMC5* KO and siINTS8, compared with the wild-type cells (log_2_FC > 1, *p*adj < 0.05). (E) Individual gene examples from TT_chem_-seq. (F) Model depicting how ARMC5 and Integrator phosphatase provide complementary mechanisms to ensure homeostasis of RNA Pol II at the early stages of the transcription cycle. See also Figure S8.

To gain further insight into how ARMC5 and INTS8 regulate RNA Pol II, we mapped total RNA Pol II occupancy using dxChIP-seq, upon depletion of *ARMC5, INTS8*, and both factors in combination. *ARMC5* KO and *INTS8* knockdown both resulted in increased levels of RNA Pol II in the promoter-proximal zone, peaking at the pausing site (Figure 7A, left), and their combination had an additive effect (Figure 7A, left). In agreement with their additive effects on Ser5^P^ levels (Figures 6D and 6G), this indicates that both ARMC5 and INTS8 act to reduce the levels of RNA Pol II in the promoter-proximal zone, likely by separate mechanisms.

Surprisingly, we noticed that RNA Pol II occupancy at gene ends has a completely inverse profile to that in the promoter-proximal zone. Loss of either ARMC5, INTS8, or both reduced the levels of RNA Pol II in the vicinity of TTSs, despite the increases in the promoter-proximal zone in these conditions (Figure 7A, right). This effect was quantified using the pausing index,^71^ which estimates the ratio of promoter-proximal vs. elongating RNA Pol II. Depletion of both *ARMC5* and *INTS8* alone increased the RNA Pol II pausing index, and their combined loss had an even greater effect (Figure 7B).

To investigate this further, we performed TT_chem_-seq to measure RNA Pol II activity. Metagene analysis revealed a global increase in nascent RNA upon *INTS8* depletion, which peaked in the first 5 kb downstream of the TSS and declined to WT levels further within gene bodies, at around 10 kb (Figure 7C). This indicates that INTS8 loss allows for excessive release of RNA Pol II into early elongation, as suggested previously,^27,34^ but these complexes do not proceed into late elongation past the 10 kb mark. Thus, a dramatic increase in the immobile RNA Pol II fraction in FRAP caused by *INTS8* depletion (Figures 6E and 6F) likely represents RNA Pol II molecules that are stably paused or are in early elongation.

Interestingly, *ARMC5* KO in combination with INTS8 loss did not further augment *INTS8*-depletion-driven global increase in early elongation activity (Figure 7C), despite having an additive effect on increasing RNA Pol II occupancy in this zone of genes (Figure 7A left). This indicates that excess RNA Pol II molecules present in the promoter-proximal zone due to loss of ARMC5, even when released into early elongation due to loss of INTS8, are even less efficiently transcribing than in the case of *INTS8* depletion alone. This suggests they may be immature or incompetent for elongation (thus giving rise to higher signal in dxChIP-seq, which measures occupancy, but not in TT_chem_-seq, which measures RNA Pol II activity). This is further supported by an observation that these excessive early elongation RNA Pol II complexes do not proceed into late elongation: in cells depleted of *INTS8, ARMC5*, and both factors in combination, both dxChIP-seq and TT_chem_-seq signals return to WT levels within 10 kb post-TSS and further decline below WT levels at gene ends, indicating that RNA Pol II must have been terminated during late elongation (Figures 7A, 7C, and S8A).

Together, these results show that both INTS8 and ARMC5 can limit the release of excess and possibly incompetent RNA Pol II complexes into late elongation, working through independent yet parallel mechanisms to control the quality and quantity of RNA Pol II on genes.

### A specific set of genes use INTS8-ARMC5 to attenuate gene expression

Since *INTS8* and *ARMC5* depletion most dramatically affect early elongation complexes (in the zone covering up to 5–10 kb from the TSS) (Figures 7A and 7C), it is possible that short and long genes may be disproportionately affected by the loss of these factors. Indeed, genes shorter than 10 kb displayed an overall increase in nascent RNA signal upon INTS8 loss, throughout the gene unit, which was surprisingly exacerbated by a combined ARMC5 loss (Figure S8B). This indicates that short genes use ARMC5 and INTS8 to prevent excessive RNA Pol II activity.

To gain a better understanding of what types of genes are co-regulated by ARMC5 and INTS8, we performed differential gene expression analysis of spike-in normalized TT_chem_-seq data (Figures 7D and S8C) and analyzed if differentially expressed genes possess any particular properties, distinguishing them from the rest of the genome. This suggested that a particular class of genes use ARMC5 and INTS8 mechanisms to attenuate their gene expression level (examples in Figure 7E): these genes tend to be short (Figure S8D) and thus below the late-elongation limit of 10 kb (at which point incompetent RNA Pol II starts being aborted); they tend to have low baseline expression (Figure S8E) and weak pausing (Figure S8F) and contain TATA boxes in their promoters (Figure S8G).

## Discussion

Here, we reveal that ubiquitylation acts to regulate RNA Pol II levels during homeostatic cell growth by targeting RNA Pol II in the transcription cycle before the transition from pausing to elongation. This RNA Pol II degradation pathway requires the Cullin-RING E3 ligase CRL3^ARMC5^, which ubiquitylates RPB1; p97/VCP, which extracts polyubiquitylated proteins from complexes^45^; and the proteasome, which degrades proteins^72^; and it is distinct from the last-resort ubiquitylation pathway that destroys RNA Pol II stalled during elongation.^5,9,41,42,46^ In the absence of this mechanism, cells are able to proliferate normally *in vitro*, and given that patients and mice carrying *ARMC5* mutations can survive to adulthood despite excess RNA Pol II,^50,52^loss of this pathway also seems to be tolerated *in vivo*. Our results suggest that this is because cells can prevent this excess RNA Pol II from entering elongation, first by keeping most RNA Pol II in the free pool, and secondly by preventing its release from the promoter-proximal region into gene bodies. However, the absence of ARMC5 makes cells strongly dependent on checkpoints at the promoter-proximal region—with concomitant loss of ARMC5 and the Integrator phosphatase module subunit INTS8 leading to a severe growth-retardation phenotype.

In yeast, RNA Pol II has been proposed to act as a ‘limiting factor’ for mRNA production that controls the coordination of mRNA synthesis rates with cell size—a key part of a mechanism of mRNA concentration homeostasis.^11^ In this model, the amount of RNA Pol II loaded on the genome depends on RNA Pol II availability. We previously proposed an alternative model, based on our human cell data, in which RNA Pol II levels are adapted to the global transcriptional activity.^4^ In this model, RNA Pol II turnover is activity dependent: it is protected from degradation when actively transcribing (stably bound to chromatin) and subject to degradation when inactive. Therefore, RNA Pol II levels diminish when absolute transcription rates are decreased. Here, we report that ARMC5 is essential for degrading RNA Pol II in response to transcriptional inhibition and that when ARMC5 is not present, RNA Pol II accumulates predominantly in the inactive, free state. Given that the CRL3^ARMC5^ pathway is a major RNA Pol II turnover pathway in unperturbed cells (Figure 2) and that ARMC5-dependent ubiquitylation is enhanced in response to diverse chemical (Figure 3) and genetic (Figure 6) perturbations, these suggest that CRL3^ARMC5^ acts quite generally and may be a key player in the mechanisms coordinating RNA Pol II levels with transcriptional activity.

Our results demonstrate that ubiquitin-dependent regulation of the cellular pool of RNA Pol II is an important element of transcriptional control—not only in the DNA-damage response, as we reported previously,^5^ but also for homeostatic control of transcription, controlled by CRL3^ARMC5^. ARMC5 removal has profound effects on global RNA Pol II homeostasis, causing accumulation of RNA Pol II in the free pool and in promoter-proximal zones of most genes in the genome (Figures 1, 2, 3, and 4). We revealed that INTS8 functions as a gatekeeper, preventing the release of these excessive transcriptional complexes into elongation, which is in agreement with its proposed function in restricting pause-release.^27,29,34^ The ability of ARMC5 and INTS8 to control the quantity of RNA Pol II on genes is exploited by a specific class of loci that uses these two mechanisms for attenuating gene expression levels: these genes are short, lowly expressed, have a low pausing index, and contain TATA boxes in their promoters. Interestingly, dependency of lowly expressed genes on suppression by both the Integrator phosphatase and endonuclease modules has already been observed in *Drosophila* and mammalian cells,^27,32^ and here, we find that these effects are further amplified by the concurrent loss of ARMC5. Furthermore, genes controlled by ARMC5 and INTS8 may be specifically involved in neuronal and T cell regulation, respectively (Figure S8H). This association may be relevant for human disease: *ARMC5* mutations increase the risk of severe neural tube defects,^51^ while *INTS8* mutations have been associated with peripheral T cell lymphoma.^73^ Together, these results support the idea that regulation of the transcription apparatus in the promoter-proximal region is likely heterogeneous across gene classes.

We also provide evidence that in addition to regulating RNA Pol II quantity on genes, both ARMC5 and INTS8 prevent the entry of incompetent transcription complexes into late elongation, acting as two parallel mechanisms with additive effects. When *ARMC5* and *INTS8* are depleted in combination, increased amounts of RNA Pol II are unleashed into early elongation, compared with removal of *INTS8* alone, but these excess RNA Pol II complexes are not fully transcriptionally active and are terminated prematurely along the gene, failing to reach gene ends. This could be because they are somehow incompetent for elongation. Given that CRL3^ARMC5^ ubiquitylates RNA Pol II when transcription complex is made ‘defective’ by a variety of chemical and genetic perturbations (Figures 3C and 6D), it is plausible that ARMC5 targets incorrectly assembled transcription complexes or those lacking key subunits. Another possibility that could explain why RNA Pol II does not reach gene ends upon *ARMC5/INTS8* depletion could be activation of yet another premature termination pathway, acting to prevent excess production of full-length transcripts in cases of elevated early elongation.

Which RNA Pol II species are directly targeted by CRL3^ARMC5^ and where in the cell they are targeted remain partially unresolved questions. It is possible that ARMC5 targets RNA Pol II predominantly in the free pool, after it has been evicted from chromatin, to control global RNA Pol II homeostasis. In this scenario, any disruption of transcription that results in RNA Pol II eviction would lead to reduced RNA Pol II levels. Elevated RNA Pol II levels in promoter-proximal regions caused by ARMC5 loss would, in this case, be a result of increased amounts of RNA Pol II in the free pool available for initiation. However, this would not directly explain why some of these excessive transcription complexes are incompetent for elongation. An alternative (and not mutually exclusive) model is that ARMC5 acts on RNA Pol II directly in the promoter-proximal region—targeting Ser5^P^ RNA Pol II as part of a checkpoint that ensures that a faulty transcription complex is terminated and targeted for degradation. To what extent faulty transcription complexes exist in unperturbed cells is unclear. However, a large number of factors assemble on RNA Pol II to orchestrate the transcription process,^2,3^ and the promoter-proximal zone is a place of complex molecular transactions, where RNA Pol II exchanges interaction partners multiple times.^68,74^ Given the complexity of these exchanges, it is possible that some of these steps may occasionally (or even often) go wrong, giving rise to incompletely or incorrectly assembled transcription complexes that are not fully capable of efficient elongation. Nonetheless, we found that RNA Pol II that is normally targeted by ARMC5 in the presence of triptolide is, in the absence of ARMC5, able to bypass inhibited XPB translocase and to proceed to transcribe into gene bodies. This suggests that at least some of the RNA Pol II normally targeted by ARMC5 is properly assembled and able to produce functional transcripts. This is supported by our observation that *ARMC5* KO cells can proliferate in low levels of triptolide that are normally lethal to cells. It is possible that ARMC5 forms a complex with different protein adapters on- and off-chromatin, targeting both defective RNA Pol II on DNA and excessive RNA Pol II in the free pool.

Altogether, we conclude that CRL3^ARMC5^ ubiquitin ligase and Integrator phosphatase form parallel mechanisms to control early stages of RNA Pol II transcription (Figure 7F). In the future, it will be interesting to identify other factors and pathways that synergize with ARMC5 to maintain transcriptional homeostasis and to define how exactly and in what circumstance ARMC5 targets RNA Pol II.

### Limitations of the study

The amount and function of RNA Pol II bound transiently to chromatin are difficult to determine. How the population of RNA Pol II shown to be chromatin-bound via fractionation relates to that observed to be immobile via FRAP is unclear. Transient interactions of RNA Pol II with DNA with intermediate timescales (up to several seconds) may appear as either mobile or immobile via FRAP, or they may appear in either chromatin-bound or soluble fractions. Whether these intermediate populations of RNA Pol II represent a paused component and how they relate to the positioning of RNA Pol II on genes via dxChIP-seq are not addressed in the current study.

Unlike higher-resolution methods such as precision run-on sequencing (PRO-seq) or native elongating transcript sequencing (NET-seq), Pol II dxChIP-seq is unable to reveal nucleotide-level RNA Pol II occupancies, which could be useful for more detailed assessment of the synergistic effects of ARMC5 and Integrator on RNA Pol II processivity and pausing.

No antibodies are available to detect endogenous ARMC5, either by western blot or immunofluorescence, which means that we rely on overexpression of ARMC5, likely well beyond physiological levels.

In conditions where RNA Pol II turnover is compromised, such as *ARMC5*-depleted cells, the extent to which the RPB1 subunit is contained in RNA Pol II complexes within the nucleus may vary substantially from unperturbed cells. Here, we broadly assume that detected RPB1 levels within the nucleus reflect levels of the RNA Pol II complex.

Finally, this study uses both CRISPR-mediated KO and siRNA-mediated knockdown to examine ARMC5 function. While the data collected using these two strategies are generally concordant, possible mechanisms counteracting ARMC5 loss (over days in knockdown condition or weeks in KO condition) are unknown and may influence the results presented here.

## Resource Availability

### Lead contact

Further information and requests for resources and reagents should be directed to and will be fulfilled by the lead contact, Ana Tufegdžić Vidaković (atv@mrc-lmb.cam.ac.uk).

### Materials availability

Materials generated in this study will be made available on request, but we may require a completed Materials Transfer Agreement if there is potential for commercial application.

### Data and code availability

- Single-cell quantifications, summaries of microscopy experiments, and western blot film scans have been deposited on Mendeley Data. Image data can be made available upon request. Data generated using high-throughput sequencing has been deposited to GEO (https://www.ncbi.nlm.nih.gov/geo/), GEO: GSE266979.
- Code used to analyze image data is available on GitHub (https://doi.org/10.5281/zenodo.14031542). Code used to analyze sequencing data is available from the lead contact upon request.

## Star+Methods

Detailed methods are provided in the online version of this paper and include the following:

- Key resources table
- Experimental model and study participant details
  - Cell lines and culture conditions
- Method details
  - Generation of *ARMC5* KO cell lines
  - Generation of mCherry-RPB1 knock-in cells
  - Cell treatments
  - Cell growth assays
  - Detection of ubiquitylated RPB1
  - Western blot
  - Chromatin fractionation
  - TT_chem_-seq (nascent RNA-seq)
  - dxChIP-seq (double-crosslinking chromatin immunoprecipitation and sequencing)
  - Preparation of cells for microscopy
  - siRNA transfection for microscopy
  - Bleach-chase experiments (protein half-life measurement)
  - Compound treatment (384-well plates, immunofluorescence and 5EU click)
  - Immunofluorescence
  - mRNA poly(A) fluorescence in situ hybridisation _B_ 5-ethynyl uridine visualisation via click chemistry _B_ Fixed cell imaging
  - Fluorescence recovery after photobleaching (FRAP)
- Quantification and statistical analysis
  - Quantitative image processing
  - Protein half-life measurement using bleach-chase
  - FRAP analysis
  - Computational analysis of genome-wide experiments

## Star+Methods

### Key Resources Table

**Table:** 

REAGENT or RESOURCE	SOURCE	IDENTIFIER
Antibodies		
Rabbit monoclonal RPB1 (total, N-terminal)	Cell Signaling	D8L4Y; RRID:AB_2687876
Mouse monoclonal RPB1 (total, N-terminal)	Santa Cruz	sc-55492, F12; RRID:AB_630203
Mouse monoclonal RPB1 (raised against S5-P, recognizes multiple forms)	Abcam	4H8; RRID:AB_304868
Rat monoclonal RPB1, phospho-serine 2	Helmholtz Zentrum Munich	3E10
Rat monoclonal RPB1, phospho-serine 5	Helmholtz Zentrum Munich	3E8
Rat monoclonal RPB1, phospho-serine 2 (UNSW)	Merck	3E10; 04-1571-I; RRID:AB_11212363
Rat monoclonal RPB1, phospho-serine 5 (UNSW)	Merck	3E8; 04-1572-I; RRID: AB_11213421
Rabbit polyclonal SPT5 (SUPT5H)	Bethyl	A300-869A; RRID:AB_609484
Mouse monoclonal Vinculin	Sigma	V9131; RRID:AB_477629
Rabbit polyclonal Histone H3	Abcam	ab18521; RRID:AB_732917
Rabbit polyclonal Gdown (GCOM1)	Proteintech	18129-1-AP; RRID:AB_2232101
Rabbit polyclonal NELFCD (TH1L)	Proteintech	11226-1-AP; RRID:AB_2201665
Rabbit polyclonal INTS8	Merck	HPA057299; RRID:AB_ 2683403
Rabbit polyclonal INTS11	Bethyl	A301-274A; RRID:AB_937779
Rabbit polyclonal PAF1	Bethyl	A300-173A; RRID:AB_2159877
Rabbit anti-mCHERRY	Abcam	ab16753; RRID:AB_2571870
Mouse monoclonal alfa-tubulin	Sigma	T6074; RRID:AB_477582
Rabbit monoclonal Flag	Cell Signaling	2368S; RRID:AB_2572291
anti-mouse secondary antibody (HRP)	Dako	P044701-2; RRID:AB_2617137
anti-rabbit secondary antibody (HRP)	Dako	P044801-2; RRID:AB_2617138
Goat anti Mouse IgG (H+L) HRP	Thermo Fisher	31430; RRID:AB_228307
Goat anti Rabbit IgG (H+L) HRP	Thermo Fisher	31460; RRID:AB_228341
anti-rat secondary antibody (HRP)	Jackson ImmunoResearch	112-035-003; RRID:AB_2338128
Goat anti-mouse Alexa488-Plus	Thermo Fisher	A32723, RRID:AB_2633275
Goat anti-rat Alexa488-Plus	Thermo Fisher	A48262, RRID:AB_2896330
Goat anti-rabbit Alexa488-Plus	Thermo Fisher	A32731, RRID:AB_2633280
Goat anti-mouse Alexa568	Thermo Fisher	A11031, RRID:AB_144696
Rabbit Anti-Rat IgG	Abcam	ab6703, RRID:AB_956015
Chemicals, peptides, and recombinant proteins		
MG-132	Cayman Chemicals	10012628
p97 inhibitor CB-5083	Stratech	S8101-SEL
MLN-4924	Tocris	6499/50
triptolide	Cayman Chemicals	CAY11973-5
JQ1	MedChemExpress	HY-13030-10mg
AZD4573	Selleckchem	S8719
flavopiridol	Santa Cruz	sc-202157A
THZ1	Apexbio	A8882
THZ531	Cayman Chemicals	26386-1
Okadaic acid	Insight Biotech.	sc-202259
N-Ethylmaleimide (NEM)	Sigma-Aldrich	E3876
4-thiouridine	Glentham Life Sciences	GN6085
4-thiouracil	Sigma-Aldrich	440736
MTSEA biotin-XX linker (2-((6-((6-((biotinoyl) amino)hexanoyl)amino)hexanoyl) amino) ethylmethanethiosulfonate)	Biotium	BT90066
Anti-Flag M2 Magnetic Beads	Sigma	M8823
Dsk2 beads	Home-made; see Tufegdzic Vidakovic et al.^46^	N/A
HRP-conjugated streptavidin	Thermo Fisher Scientific	N100
Lipofectamine 3000	Thermo Fisher Scientific	L3000015
High glucose DMEM	Thermo Fisher Gibco	31966047
Poly-lysine	Sigma-Aldrich	P7280
4 to 12% Tris-Glycine Plus Protein Gels	Invitrogen	WXP41226BOXA
Complete EDTA-free protease inhibitor cocktail	Sigma-Aldrich	05056489001
cOmplete Protease Inhibitor Cocktail	Roche	11697498001
PhosSTOP	Sigma-Aldrich	04906837001
Nitrocellulose membrane	GE Healthcare Life Sciences	10600002
Nitrocellulose membrane	Thermo Fisher Scientific	STM2007
SuperSignal West Pico PLUS ECl reagent	Thermo Fisher Scientific	34577
Radiance Plus ECL	Azure Biosystems	AC2103
Benzonase	MerckMillipore	70746-4
TRIzol Reagent	Thermo Fisher Scientific	15596026
Triptolide (UNSW)	Sapphire Bioscience, Adipogen	AG-CN2-0448-M001
THZ1 2HCl (UNSW)	Sapphire Bioscience, Selleckchem	S7549
AZD4573 (UNSW)	Sapphire Bioscience, Selleckchem	S8719
LDC-4297 (UNSW)	Sapphire Bioscience, Cayman Chemical	23398
5,6-Dichlorobenzimidazole 1-β-D-ribofuranoside (DRB) (UNSW)	Merck Sigma Aldrich	A2263
Dithiobis (succinimidyl propionate) (DSP)	Thermo Fisher	22585
CB-5083 (UNSW)	Focus Biosciences, MedChemExpress	HY-12861
Alexa Fluor 647 NHS ester	Thermo Fisher Invitrogen	A20006
Alexa Fluor 488 NHS ester	Thermo Fisher Invitrogen	A20000
Alexa Fluor 647 azide	Thermo Fisher Invitrogen	A10277
Sodium ascorbate	Sigma Aldrich	A7631
Copper sulphate	Chem-Supply Australia	CA061
5-ethynyl uridine (5-EU)	Lumiprobe	2439
DAPI (4’,6-diamidino-2-phenylindole, dihydrochloride)	Thermo Fisher	D1306
DMEM, high glucose, pyruvate	Thermo Fisher Gibco	11995065
Fetal bovine serum	Moregate Biotech	N/A
McCoy’s 5A (modified)	Thermo Fisher Gibco	16600108
McCoy’s 5A, no phenol red	Cytiva	SH30270.01
Opti-MEM	Thermo Fisher Gibco	31985-062
Penicillin-streptomycin	Sigma Aldrich	P0781
Paraformaldehyde	EMS Emgrid	15710
Triton X100	Sigma Aldrich	93443
Formamide	Thermo Fisher Invitrogen	AM9342
Saline sodium citrate buffer	Thermo Fisher Invitrogen	AM9763
Ribonucleoside vanadyl complexes	New England Biolabs	S1402S
Yeast transfer RNAs	Thermo Fisher Invitrogen	15401011
Ultra-pure BSA	Thermo Fisher Invitrogen	AM2616
Dextran sulfate	Merck Sigma Aldrich	D8906-50G
Lipofectamine RNAiMAX transfection reagent	Thermo Fisher Invitrogen	13778100
Tris pH 8	Thermo Fisher Invitrogen	AM9856
DSG (disuccinimidyl glutarate)	Thermo Scientific	20593
16% Formaldehyde (w/v), Methanol-free	Thermo Scientific	28908
Glycine	Fisher Chemical	G/0800/60
Proteinase K	Invitrogen	AM2546
Critical commercial assays		
RNA minElute clean-up kit	QIAGEN	74204
μMACS Streptavidin Kit	Miltenyi	130-074-101
KAPA RNA HyperPrep Kit	Kapabiosystems	KR1350
Micro Bio-Spin P-30 Gel Columns	BioRad	7326223
Qubit protein assay kit	Invitrogen	Q33212
Qubit dsDNA High Sensitivity assay kit	Invitrogen	Q33230
Qubit RNA assay kit	Invitrogen	Q32852
High Sensitivity DNA ScreenTape Analysis	Agilent	5067-5593, 5067-5592
PureLink RNA Mini kit	Invitrogen	12183018A
Protein G Dynabeads	Fisher Scientific	10004D
ChIP DNA Clean & Concentrator	Zymo Research International	D5205
NEBNext Ultra II DNA Library Prep Kit	NEB	E7645L
MaXtract High Density tubes	QIAGEN	129056
Bioanalyzer High Sensitivity RNA Analysis	Agilent	5067-1513
Deposited data		
Genome-wide sequencing data are available under GEO number GSE266979	This paper	GEO: GSE266979
Image quantification: mCherry-RPB1 bleach-chase	This paper	Mendeley Data:https://doi.org/10.17632/rvm2sxs7br.1
Image quantification: mCherry-RPB1 FRAP	This paper	Mendeley Data:https://doi.org/10.17632/427d6wcxb5.1
Image quantification: RPB1 immunofluorescence in HEK293 ARMC5 KO cells	This paper	Mendeley Data:https://doi.org/10.17632/y87g8mb2z3.1
Image quantification: RPB1 immunofluorescence in HCT116 cells (ARMC5, INTS8 siRNA)	This paper	Mendeley Data:https://doi.org/10.17632/42hcnbdr4t.1
Image quantification: Poly(A) FISH in HEK293 ARMC5 KO cells	This paper	Mendeley Data:https://doi.org/10.17632/hhd88xm56z.1
Image quantification: 5-EU in HEK293 ARMC5 KO cells	This paper	Mendeley Data:https://doi.org/10.17632/fwn9z2j3kz.1
Image quantification: RPB1 immunofluorescence in HEK293 ARMC5 KO cells with transcriptional inhibitors	This paper	Mendeley Data:https://doi.org/10.17632/6h3npxnmv2.1
Image quantification: RPB1 immunofluorescence in HCT116 cells (ARMC5 siRNA) with transcriptional inhibitors	This paper	Mendeley Data:https://doi.org/10.17632/8n7zcmzrbn.1
5-Ethynyl Uridine nascent RNA labelling in HCT116 cells (ARMC5 siRNA)	This paper	Mendeley Data:https://doi.org/10.17632/zwccwsynk8.1
Scans of Western Blot films	This paper	Mendeley Data:https://doi.org/10.17632/5z5x7349cc.1
Experimental models: Cell lines		
Flp-In T-Rex HEK293 cells	Thermo Fisher Scientific	R78007
RPB1 K1268R knock-in clone D12 (in Flp-In T-Rex HEK293)	Tufegdžić Vidakovic et al.^5^	N/A
ARMC5 knock-out clone 1A3 (in Flp-In T-Rex HEK293)	This paper	N/A
ARMC5 knock-out clone 2B6 (in Flp-In T-Rex HEK293)	This paper	N/A
HCT116	ATCC	CCL-247
HCT116 mCherry-RPB1 clone 2 (in HCT116)	This paper	N/A
Oligonucleotides		
Poly(A) FISH probe: dT-30-ATTO647N	IDT	N/A
siRNAs targeting ARMC5, Silencer Select	Thermo-Fisher	s36352, s36353, s229821
siRNAs targeting INTS8, Silencer Select	Thermo Fisher	s31179, s31180, s31181
ON-TARGETplus Non-targeting Control Pool siRNA	Dharmacon	D-001810-10
ON-TARGETplus SMARTpool siRNA targeting human Gdown	Dharmacon	L-007919-01
ON-TARGETplus SMARTpool siRNA targeting human SPT5	Dharmacon	L-016234-00
ON-TARGETplus SMARTpool siRNA targeting human NELFCD	Dharmacon	L-020811-01
ON-TARGETplus SMARTpool siRNA targeting human INTS8	Dharmacon	L-020270-02
ON-TARGETplus SMARTpool siRNA targeting human INTS11	Dharmacon	L-013789-01
ON-TARGETplus SMARTpool siRNA targeting human PAF1	Dharmacon	L-020349-01
ON-TARGETplus SMARTpool siRNA targeting human Cullin 1	Dharmacon	L-004086-00
ON-TARGETplus SMARTpool siRNA targeting human Cullin 2	Dharmacon	L-007277-00
ON-TARGETplus SMARTpool siRNA targeting human Cullin 3	Dharmacon	L-010224-00
ON-TARGETplus SMARTpool siRNA targeting human Cullin 4A	Dharmacon	L-012610-00
ON-TARGETplus SMARTpool siRNA targeting human Cullin 4B	Dharmacon	L-017965-00
ON-TARGETplus SMARTpool siRNA targeting human Cullin 5	Dharmacon	L-019553-00
ON-TARGETplus SMARTpool siRNA targeting human Cullin 7	Dharmacon	L-017673-00
ON-TARGETplus SMARTpool siRNA targeting human Cullin 9	Dharmacon	L-014128-00
Figure S1B primer inside F CTCAGCATCCTAGCCGATTG	This paper	N/A
Figure S1B primer inside R CGTTATTCCGGGATAGGACA	This paper	N/A
Figure S1B primer outside F TTCCGGACTTTGTGACTGTG	This paper	N/A
Figure S1B primer outside R CTGTGTGTCCAGTTGGGTTG	This paper	N/A
Figure S1B gRNA 1F for ARMC5 KO CACCGCTAAAAGCCTTACCGCTGAG	This paper	N/A
Figure S1B gRNA 1R for ARMC5 KO AAACCTCAGCGGTAAGGCTTTTAGC	This paper	N/A
Figure S1B gRNA 2F for ARMC5 KO CACCGAGCAGAAGGAGTCATCATGG	This paper	N/A
Figure S1B gRNA 2R for ARMC5 KO AAACCCATGATGACTCCTTCTGCTC	This paper	N/A
Figure S2C primer RPB1 F (-430 from ATG) TCTATAAGAAGCGTCGTTCAGC	This paper	N/A
Figure S2C primer RPB1 R (+349 from ATG) AATCAGTCATCCTTCTCTCCCT	This paper	N/A
Recombinant DNA		
pSpCas9n(BB)-2A-GFP-gRNAs	This paper	N/A
Donor plasmid for ARMC5 KO	This paper	N/A
mAC-POLR2A donor (Hygro)	Gift from Masato Kanemaki; Nagashima et al.^75^	RRID:Addgene_124496
POLR2A-N CRISPR pX330	Gift from Masato Kanemaki; Nagashima et al.^75^	RRID:Addgene_124495
pRRL_U2AF1_WT_mCherry	Gift from Robert Bradley; Ilagan et al.^76^	RRID:Addgene_84017
mCherry-POLR2A donor	This paper	N/A
ARMC5-Flag	GeneCopoeia	EX-H0661-M35
Software and algorithms		
Trim Galore V0.6.7	Babraham Bioinformatics	https://github.com/FelixKrueger/TrimGalore
Bowtie2 V8.3.1	Langmead et al.^77^; Langmead and Salzberg^78^	https://github.com/BenLangmead/bowtie2
SAMtools V1.9	Li et al.^79^	https://www.htslib.org/
Picard V2.27.5	Broad Institute	http://broadinstitute.github.io/picard
DeepTools V3.5.1	Ramirez et al.^80^	https://github.com/deeptools/deepTools
BEDtools V2.30.0	Quinlan and Hall^81^	https://bedtools.readthedocs.io/en/latest/
dplyr	Wickham et al.^82^	https://cran.r-project.org/web/packages/dplyr/index.html
ggplot2	Wickham^83^	https://cran.r-project.org/web/packages/ggplot2/index.html
STAR v2.7.9a	Dobin et al.^84^	https://github.com/alexdobin/STAR
HTSeq V2.0.5	Anders et al.^85^	https://github.com/simon-anders/htseq
DESeq2	Love et al.^86^	https://bioconductor.org/packages/release/bioc/html/DESeq2.html
eulerr	Larsson^87^	https://github.com/jolars/eulerr
clusterProfiler	Wu et al.^88^; Yu et al.^89^	https://github.com/YuLab-SMU/clusterProfiler
scikit-image	van der Walt et al.^90^	https://pypi.org/project/scikit-image/
mahotas	Coelho^91^	https://github.com/luispedro/mahotas
blimp	This paper	https://doi.org/10.5281/zenodo.12559364
Other		
UV radiometer	Vilber	VLX-3W, SX254

### Experimental Model And Study Participant Details

#### Cell lines and culture conditions

Wild-type, *ARMC5* KO, and K1268R Flp-In T-REx HEK293 (R78007, Thermo Fisher Scientific) cell lines were cultured in supplemented high glucose DMEM (31966021, Thermo Fisher Scientific) with 10% foetal bovine serum (FBS), 100 U/mL penicillin, 100 μg/mL streptomycin at 37 °C with 5% CO_2_. RPB1 K1268R mutant HEK293 cell line was generated in an earlier study.^5^ For microscopy experiments, HCT116 and HCT116 mCherry-RPB1 cells were maintained in McCoy’s 5A modified medium (Thermo Fisher Gibco 16600108), and HEK293 parental and *ARMC5* knock-out cells were maintained in Dulbecco’s Modified Eagle Medium containing high glucose (Thermo Fisher Gibco 11995065), both in 10% FBS (Moregate Biotech) at 37 ºC and 5% CO_2_. HEK293, and cell lines derived from these, are female. HCT116 cells, and cell lines derived from these, are male. Cells were not authenticated.

### Method Details

#### Generation of *ARMC5* KO cell lines

CRISPR-Cas9 mediated genome editing of Flp-In T-REx HEK293 cell lines was performed as previously described.^92^ The oligonucleotides encoding gRNAs for targeting *ARMC5* locus are listed in the Key Resources Table. Briefly, the forward and reverse strand oligonucleotides were annealed and ligated into pSpCas9(BB)-2A-GFP linearized with BbsI, and plasmids were sequenced after cloning and transformation. To generate knock-outs, cells were co-transfected with the two pSpCas9(BB)-2A-GFP plasmids containing gRNA 1 and 2 using Lipofectamine 3000 (Thermo Fisher Scientific) according to the manufacturer’s instructions. 24 h after transfection, high GFP-positive cells were sorted clonally by FACS into 96-well plates and cultivated until colonies were obtained. Clones were tested for deletion of the entire *ARMC5* locus by genotyping, with primers flanking the gene and primers within the gene (Figure S1A).

#### Generation of mCherry-RPB1 knock-in cells

A CRISPR homology-directed repair donor plasmid encoding mCherry-RPB1 was generated by Gibson assembly. Assembly fragments were amplified by PCR from a mAID-mClover-POLR2A donor plasmid^75^ (Addgene #124496) and mCherry-U2AF1 plasmid^76^ (Addgene #84017) templates. The assembled mCherry-RPB1 donor plasmid contains a left homology arm from -530bp to 0bp upstream of the start codon, followed by the mCherry fusion protein, a Gly-Ala-Gly-Ala-Gly-Ala-Gly-Ser linker, then a 584bp right homology arm, including the endogenous ATG from RPB1. No additional tags or antibiotic resistance markers are present.

Cells were transfected with this mCherry-RPB1 donor plasmid and a pX330 Cas9 gRNA plasmid previously used to generate miniAID-mClover-POLR2A cells^75^ (Addgene #124495). Upon transfection, mCherry was expressed transiently from the plasmid for several days, as inferred from cytoplasmic fluorescence. After 5-7 days, this cytoplasmic signal disappeared and a lower level of nuclear mCherry signal was detected in a subpopulation of cells. To isolate these cells, we used fluorescence-activated cell sorting (FACS) to sort single cells into individual wells of a 96-well plate, keeping only those with the highest 5% of mCherry signal. These were manually validated as single cells and expanded to generate putative mCherry-RPB1 clones. Clones were genotyped by PCR (Figure S2D), genomic DNA was Sanger sequenced (Figure S2E), and expression of full-length tagged protein was validated by western blot (Figure S2F). All experiments were performed with a single homozygous clone (clone 2). RPB1 abundance is similar between untagged and mCherry-tagged cell lines (Figures S2F and S2G).

#### Cell treatments

For TT_chem_-seq, cell growth and Dsk2 pulldown assays, siRNA transfections were performed with Lipofectamine RNAiMax (Thermo Fisher) according to manufacturer instructions, with 40 nM final siRNA concentration. UV irradiation was performed using a custombuilt UV conveyor belt and the given dose was determined using a UV-meter.^46^ MG-132, p97 inhibitor CB-5083, MLN-4924, triptolide, THZ1, JQ1, AZD4573, flavopiridol, THZ531, LDC4297, DRB, and okadaic acid were used as indicated in figures or figure legends and are listed at key resources table.

#### Cell growth assays

For analysis of cell growth, 5,000 HEK293 cells were seeded per well in poly-lysine (P7280, Merck) coated 96-well plates 24 hours after siRNA transfections. Growth was monitored and recorded every 4 h using Incucyte (Sartorius). Data from one representative experiment of three biological replicates, each with 5 technical replicate wells per condition and 4 imaging areas per each well, were used for plotting.

#### Detection of ubiquitylated RPB1

Whole cell lysates were prepared by scraping the cells in PBS, spinning down at 300 rcf and removing the supernatant. Cell pellet was resuspended in TENT buffer (50 mM Tris-HCl pH 7.4, 2 mM EDTA, 150 mM NaCl, 1% Triton X-100) containing fresh protease inhibitors, phosphatase inhibitors and 2 mM of N-ethylmaleimide (NEM). Samples were incubated on ice for 10 min, sonicated in a 4 °C water bath sonicator (Bioruptor) at high power, with 30 s ON and 30 s OFF pulses, for a total duration of 7 min, then centrifuged at maximum speed (18,000 rcf) for 7 min to remove debris. GST-Dsk2 pulldown of ubiquitylated proteins in human cells has been previously described in detail.^46^ Dsk2 beads were pre-washed in TENT buffer containing fresh protease inhibitors, phosphatase inhibitors and 2 mM NEM. A bead suspension of 0.2–0.4 mL (equivalent to 10–20 μL packed beads) was used to pull down ubiquitylated proteins from 1–2 mg of the whole cell protein extract. Samples were incubated on a turning wheel at 4 °C overnight. The beads were then washed twice with 1 mL of TENT buffer containing fresh protease inhibitors, phosphatase inhibitors and 2 mM NEM, and then once with 1 mL of PBS containing protease inhibitors, phosphatase inhibitors and 2 mM NEM. The samples were then centrifuged at 500 rcf for 5 min at 4 °C, all liquid was removed, and 40 μL of Laemmli buffer containing DTT were added to the beads. Samples were vortexed briefly, boiled at 98 C for 5 min, spun down and supernatants were saved and analysed by Western Blot.

#### Western blot

For whole cell extracts, cells pellets were resuspended in protein lysis buffer (20 mM Tris-HCl pH 7.5, 250 mM NaCl, 1 mM EDTA, 0.5% (v/v) NP-40, 10% glycerol, supplemented with protease inhibitors, phosphatase inhibitors and 2 mM NEM) and then sonicated in a 4 °C water bath sonicator (Bioruptor) at high power, with 30 s ON and 30 s OFF pulses, for a total duration of 7 min, then centrifuged at maximum speed (18,000 rcf) for 7 min to remove debris. Protein concentration was measured using Qubit protein assay (Q33212, Thermo Fisher Scientific) and normalised with protein lysis buffer. Proteins were separated on 4%–12% or 4%-20% Tris-Glycine gels (Thermo Fisher Scientific) and transferred to nitrocellulose membranes (Amersham). Membranes were blocked in 5% (w/v) skimmed milk in PBST (PBS, 0.1% (v/v) Tween-20) for 1 h at room temperature and incubated with primary antibody (in 5% (w/v) skimmed milk in PBST) overnight at 4 °C. Primary antibodies are listed in Key Resources Table. Membranes were subjected to 3 rinses and 3 x 5 min washes with PBST, incubated in 5% (w/v) skimmed milk in PBST containing HRP-conjugated secondary antibody (Key Resources Table), and visualised using either Radiance plus Chemiluminescent Substrate ECL reagent (Azure Biosystems) or SuperSignal West Pico PLUS (Thermo Fisher Scientific). To assess the relative abundance of RPB1 in distinct cellular fractions (soluble proteins, chromatin-associated proteins, and whole-cell lysate) between WT and *ARMC5* KO strains, western blot quantification was performed. Band intensities corresponding to RPB1 were quantified using ImageJ (version 1.53k). Relative intensities of RPB1 bands were normalized using the band intensities of Vinculin for soluble protein fractions and whole-cell lysate, or histone H3 for chromatin-associated proteins.

#### Chromatin fractionation

The cells were scraped in PBS, span down and the pellet was snap-frozen in liquid nitrogen. The frozen pellets were defrosted at room temperature and transferred to ice. 265 μL of soluble extraction buffer (20 mM HEPES-KOH pH=7.5, 150 mM potassium acetate, 1.5 mM MgCl_2_, 10% v/v glycerol, 0.05% NP-40, with addition of fresh protease inhibitors, phosphatase inhibitors and 2 mM NEM) were used to resuspend each cell pellet, and the suspensions were incubated on ice for 20 minutes. To release the cytosol and nucleoplasm, 20 strokes with a loose micropestle were applied to each sample. The chromatin was pelleted by centrifugation at 1000 rcf at 4ºC for 10 minutes and the supernatant kept as soluble fraction. The pellets were washed with soluble extraction buffer, resuspended in 100 μL of chromatin extraction buffer 1 (125 U/mL of benzonase in 20 mM HEPES-KOH pH=7.5, 1.5 mM MgCl_2_, 10% glycerol, 150 mM NaCl, 0.05% NP-40, with addition of fresh protease inhibitors, phosphatase inhibitors and 2 mM NEM) and incubated on ice for 30 minutes. Each sample was centrifuged at 20,000 rcf at 4ºC for 10 minutes and the supernatants saved as the first fraction in a new tube. The pellets were resuspended in 50 μL of chromatin extraction buffer 2 (20 mM HEPES-KOH pH=7.5, 1.5 mM MgCl_2_, 10% glycerol, 3 mM EDTA, 500 mM NaCl, 0.05% NP-40, with addition of protease inhibitors, phosphatase inhibitors and 2 mM NEM) and incubated on ice for 10 minutes with occasional gentle vortexing. The samples were centrifuged at 20,000 rcf at 4ºC for 5 minutes and the supernatant transferred to a new tube as the second chromatin fraction, to which 115 μL of chromatin dilution buffer (20 mM HEPES-KOH pH=7.5, 1.5 mM MgCl_2_, 10% glycerol, 3 mM EDTA, 0.05% NP-40, with addition of protease inhibitors, phosphatase inhibitors and 2 mM NEM) were added. These diluted second fraction samples were further centrifuged at 20,000 rcf at 4ºC for 5 minutes. The resulting supernatants were combined with the corresponding first chromatin fractions - giving rise to 265 μl of each total chromatin fraction. For Western blot analysis, protein concentration was determined using Qubit, and equal volumes of soluble and chromatin fractions was ran on 4%–12% or 4%-20% Tris-Glycine gels (Thermo Fisher Scientific), whereby samples in each fraction were normalised to the lowest-concentration sample in the set, to represent nearly equal number of cells in each well.

#### TT_chem_-seq (nascent RNA-seq)

TT_chem_-seq was performed essentially as described,^67^ with minor modifications.^5^ For each condition, 2 wells of a 6-well plate were seeded, each containing 6 x 10^5^ cells, and transfected with siRNAs (Dharmacon, L-020270-02 and D-001810-10) the next day, using Lipofectamine RNAiMax (Invitrogen, 13778100), according to manufacturer’s instructions. 40 nM final siRNA concentration was used. 24 hours after transfection, cells of the same condition were re-seeded and combined into a single 10 cm dish. The following day, cells were treated with triptolide or vehicle, and nascent RNA was *in vivo* labelled with a 1 mM 4sU (Glentham Life Sciences, GN6085) pulse for exactly 15 min (e.g. for 2-hour triptolide time point samples, 4sU was added 1 h and 45 min after treatment). Labelling was stopped by TRIzol (Thermo Fisher Scientific, 15596026) and RNA extracted as described previously.^67^

As a control for sample preparation, *S. cerevisiae* (strain BY4741, MATa, his3D1, leu2D0, met15D0, ura3D0) 4-thiouracil (4TU)-labelled RNA was spiked in to each sample. *S. cerevisiae* were grown in YPD medium overnight, diluted to an OD_600_ of 0.1, and grown to mid-log phase (OD_600_ of 0.8) and incubated with 5 mM 4TU (Sigma-Aldrich, 440736) for 6 min. Yeast can metabolise 4TU and produce 4sU, which gets incorporated into the nascent RNA. Total yeast RNA was extracted using the PureLink RNA Mini kit (Thermo Fisher Scientific, 12183020) following the enzymatic protocol.

For purification of 4sU labelled RNA, 100 μg of human 4sU-labelled RNA was spiked-in with 1 μg of 4sU-labelled *S. cerevisiae* RNA. The 101 μg of RNA (in a total volume of 100 μL) were fragmented by addition of 20 μL freshly made 1 M NaOH and incubated on ice for 20 min. Fragmentation was stopped by addition of 80 μL 1 M Tris pH 6.8 and the samples cleaned up twice with Micro Bio-Spin P-30 Gel Columns (BioRad, 7326223) adding 200 μL of RNA solution per column. The biotinylation of 4sU-residues was carried out in a total volume of 250 μl, containing 10 mM Tris-HCl pH=7.4, 1 mM EDTA and 5 μg MTSEA biotin-XX linker (Biotium, BT90066) for 30 min at room temperature in the dark. The RNA was then purified by phenol:chloroform extraction, denatured by 10 min incubation at 65 °C and added to 200 μL of μMACS Streptavidin MicroBeads suspension (Milentyl, 130-074-101). The RNA was incubated with the beads for 15 min at room temperature and the mix was applied to a pre-equilibrated μColumn in the magnetic field of a μMACS magnetic separator. Beads were washed twice with wash buffer (100 mM Tris-HCl pH=7.4, 10 mM EDTA, 1 M NaCl and 0.1% Tween20). Bio-tinylated RNA was eluted twice by addition of 100 mM DTT and cleaned up withRNeasy MinElute kit (QIAGEN, 74204) using 1050 μL of 100% ethanol per 200 μL reaction after addition of 700 μL RLT buffer to also bind short RNA fragments to the silica matrix.

Libraries for RNA sequencing were prepared using the KAPA RNA HyperPrep Kit (KR1350) with modifications. 75 ng of RNA per sample were mixed with FPE Buffer, but fragmentation procedure was omitted and RNA was instead denatured at 65 °C for 5 min. The rest of the procedure was performed as recommended by the manufacturer, with the exception of SPRI bead purifications: after adapter ligation, 0.95x and 1x SPRI bead-to-sample volume ratios were used (instead of two rounds of SPRI purification with 0.63x volume ratios). This was done to retain smaller (150-300 bp) cDNA fragments in the library which would otherwise be lost in size selection. The libraries were quality controlled by electrophoresis on a Tapestation system (Agilent), quantified by Qubit (Thermofisher), pooled and sequenced with single end 70 bp reads on a NextSeq2000, with 50,000,000 average reads per sample. Biological triplicates were generated for each condition.

#### dxChIP-seq (double-crosslinking chromatin immunoprecipitation and sequencing)

Two 15 cm dishes were seeded per condition, each containing 8.4 x 10^6^ cells. The following day, cells were treated with DMSO or 300 nM Triptolide for 2 h, the media was then removed and the cells were quickly washed twice with PBS. After the final wash, 12 mL of 1.66 mM disuccinimidyl glutarate (DSG) in PBS were added to each plate and incubated at room temperature for 15 min. The DSG solution was then removed, the cells were quickly washed with PBS three times, and 11 mL of freshly prepared solution containing 1% formaldehyde in PBS with 5 mM HEPES-KOH pH 7.5, 10 mM NaCl, 0.1 mM EDTA, 50 μM EGTA were added to each plate. After 8 minutes of incubation at room temperature, the reaction was quenched with 1 mL of 1.25 M glycine. After 5 minutes, the cells were washed 3 times with ice-cold PBS, scraped and centrifuged at 2,000 rcf at 4ºC for 7 minutes. The pellet was snap-frozen in liquid nitrogen and stored at -70°C.

The pellets were quickly thawed at room temperature, resuspended in LB1 buffer (50 mM HEPES-KOH pH=7.5, 140 mM NaCl, 1 mM EDTA, 10% Glycerol, 0.5% NP-40, 0.25% Triton X-100, with addition of protease inhibitors, phosphatase inhibitors and 2 mM NEM) and incubated for 20 minutes while rotating at 4ºC. The cells were then centrifuged at 1,000 rcf at 4ºC for 5 minutes. Each pellet was resuspended in 20 mL of LB2 buffer (10 mM Tris HCl pH=8, 200 mM NaCl, 1 mM EDTA, 0.5 mM EGTA, protease inhibitors, phosphatase inhibitors, 2 mM NEM), incubated for 5 minutes at 4ºC and centrifuged at 1,000 rcf at 4ºC for 5 minutes. The pellets containing the chromatin were then resuspended in 2mL of LB3 (10 mM Tris HCl pH=8, 100 mM NaCl, 1 mM EDTA, 0.5 mM EGTA, 0.1% freshly added Na-Deoxycholate, 0.5%, N-lauroylsarcosine, with freshly added phosphatase inhibitors, protease inhibitors and 2 mM NEM) and transferred to 1mL Covaris tubes (Covaris, 520135). Chromatin shearing was performed using a Co-varis E220 for 4 minutes with the following settings: PIP=150, CPB=1000, Duty factor=20%, Temperature=5ºC).

Sheared chromatin was transferred to 2 mL tubes and 200 μL of 10% Triton X-100 was added and mixed into each sample before centrifuging at 20,000 rcf at 4ºC for 20 minutes. Supernatant was transferred to a new tube and pre-cleared with protein G dyna-beads, in 0.1% BSA solution in PBS, for 1 h at room temperature. Beads were separated from the chromatin using a magnetic separator, and supernatant (pre-cleared chromatin) was transferred to a new protein-LoBind Eppendorf tube (0030108116). An aliquot of the pre-cleared chromatin was reserved to use as input and the rest was used for immunoprecipitation. For immunoprecipitation, 50 μL protein G dynabeads (10004D, Fisher Scientific) per sample were pre-washed and pre-coated with the desired antibody. Pre-coating was done by incubating the beads with the 20 μg of antibodies (Pol II D8L4Y, RRID:AB_2687876 and Ser5^P^ Pol II, 3E8, Helmholtz Zentrum Munich) resuspended in BSA-PBS solution, for 1h at room temperature, then washing the antibody-conjugated beads two times with BSA-PBS solution, and resuspending in LB3. Antibody detecting Ser5^P^ Pol II is derived from rat, thus for this condition the beads were pre-coated with 30 μg rabbit anti-rat IgG (ab6703, Abcam), washed three times with BSA-PBS solution, and then conjugated with anti-Ser5^P^ Pol II antibody. Antibody conjugated beads were added to chromatin and the samples were incubated overnight at 4ºC on a turning wheel. The next day, the beads were washed 5 times with ice-cold RIPA buffer (50 mM HEPES-KOH pH=7.5, 500 mM LiCl, 1 mM EDTA, 1% NP-40, 0.7% freshly added Sodium Deoxycholate) and eluted with elution buffer (25 mM Tris-HCl pH=7.5, 5 mM EDTA, 0.5% SDS) at 65ºC for 1 hour with shaking. The supernatant is transferred to a new tube and treated with Proteinase K (AM2546, Invitrogen) overnight at 60ºC. The DNA is purified with silica columns (D5205, Zymo Research) and libraries prepared with NEBNext Ultra II DNA Library prep kit (E7645L, NEB). The libraries were sequenced with paired end 60 bp reads on a NextSeq2000, with 30,000,000 average reads per sample. Biological triplicates were generated for each condition.

#### Preparation of cells for microscopy

Microscopy compatible clear plastic 96-well plates (Greiner μClear 781091), 384-well plates (Greiner μClear 781091), or 8-well glass-bottom chamber slides (ibidi 80827) were coated with poly-L-lysine (Sigma Aldrich P1399) at 100 μg/mL for 1 hour, washed twice with PBS, residual volume aspirated and allowed to dry before cells were plated. Parental or ARMC5 knock-out HEK293 cells were plated on 384-well plates at 2500 cells per well, in DMEM (Thermo Fisher Gibco 11995065) + 10% FBS (Moregate Biotech). HCT116 or HCT116 mCherry-RPB1 cells were plated on 96-well plates at 5000 cells per well, on 384-well plates at 1500 cells per well (both plates without poly-L-lysine coating), and in 8-well chamber slides at 8000 cells per well (with poly-L-lysine coating), in McCoy’s 5A (Thermo Fisher Gibco 16600108) + 10% FBS (Moregate Biotech). Cells were cultured for three days before imaging.

#### siRNA transfection for microscopy

siRNA transfections were performed as previously described.^4^ In 96-well plates, 25 μL of siRNA at 30 nM in Opti-MEM (Thermo Fisher 31985-062) was added per well, followed by 25 μL of transfection reagent (Thermo Fisher Lipofectamine RNAiMAX 13778100) diluted 1/125 in OptiMEM. In 384-well plates, 10 μL of siRNA at 30 nM in Opti-MEM was added per well, followed by 10 μL of diluted trans-fection reagent. In 8-well chamber slides, 40 μL of siRNA at 30 nM in OptiMEM was added per well, followed by 40 μL of diluted transfection reagent. After 20–30 minutes of room temperature incubation, cells were added onto the transfection reaction and allowed to settle. Experiments were conducted three days after siRNA transfection.

With the exception of Figure S6D, all microscopy siRNA experiments used pools of 3 siRNAs, with the amount of each individual siRNA reduced to maintain the total siRNA concentration as a constant. In experiments where two pools were combined (Figure 6), half concentrations of individual pools were used for comparison, with the remaining fraction made up with scrambled control siRNA.

#### Bleach-chase experiments (protein half-life measurement)

mCherry-RPB1 half-life measurements were made using the ‘bleach-chase’ method.^53^ This involves partially bleaching cells expressing a fluorescent protein and monitoring the rate of fluorescence recovery over time to infer protein turnover dynamics (further details in quantification and statistical analysis).

Imaging was performed on a Nikon Ti2 microscope with CSU-W1 spinning disk 72–90h after siRNA transfection, using a 20X/ 0.75NA objective and Hamamatsu ORCA-Fusion C14440-20UP camera (image pixel size 325nm x 325nm). The entire imaging experiment was automated using Nikon JOBS. Pre- and post-bleach imaging used 561nm laser excitation and a 617/73 nm bandpass emission filter, for ‘bleached’ and ‘unbleached control’ regions with the same well (9 imaging sites each). Bleach steps were performed using a widefield light source, from a mercury vapour lamp with 635/60 nm filter. In all cases, seven z-planes were acquired with a spacing of 2.0 μm. Two wells (ARMC5 siRNA and scrambled siRNA) were imaged sequentially for each timepoint. Allowing 10 min between frames resulted in a time between frames of approximately 14.4 min. Images were acquired for 6 frames before the bleach step and recovery was monitored for 9 h after the bleach step. The mean loss of mCherry-RPB1 intensity induced by the bleach pulse was 43 ± 1% – optimised to minimise bleach pulse duration and to retain the ability to visualise cells using mCherry signal after bleaching. Video S1 shows an example time-course.

Experiments were repeated on three different days, with two replicates performed each day. Cell confluency was monitored post hoc by examining the change in nuclear area distributions over time (nuclear area decreases with cell number as cultures become close to confluent). Wells that showed a decrease in mean nuclear area with cell number during post-bleach acquisition were excluded, due to a confounding effect of cell morphology changes on mean fluorescence intensity measurements and the possibility of changes in cell growth rate at confluency. After excluding these data, four experimental replicates were obtained, across three repeats of the experiment on different days. Each replicate consisted of 6500-16000 quantified cells.

#### Compound treatment (384-well plates, immunofluorescence and 5EU click)

3 days after plating cells, media was changed via a 2x wash to either McCoy’s 5A (HCT116) or DMEM (HEK293) media containing 10% FBS and 1% penicillin-streptomycin (Sigma Aldrich P0781). All compounds were initially dissolved at 25 mM in DMSO, aliquoted and stored at -80 ºC.

For immunofluorescence, compounds were added in 20 μL of media at 5x concentrations onto 80 μL, for a consistent final 0.4% DMSO vehicle, at indicated timepoints.

For the EU nascent RNA assay, compounds were added at 5x concentrations in 15 μL onto 60 μL at indicated timepoints. 5-ethynyl uridine (Lumiprobe 2439) was then added at 600 μM in 15 μL onto 75 μL, containing either the relevant compound or vehicle at 1x concentration, for 100 μM final EU at the indicated timepoint with maintained vehicle and compound concentrations.

#### Immunofluorescence

All steps were followed by three PBS washes. Cells were fixed in 4% paraformaldehyde (EMS Emgrid 15710) for 15 minutes, then permea-bilised in 0.25% Triton X100 (Sigma Aldrich 93443) for 10 minutes. Cells were incubated in 50% blocking buffer (Millenium Biosciences Li-Cor Intercept in PBS, LCR-927-70001) in PBS for 30 minutes, before being stained with primary antibodies in 50% blocking buffer in PBS for 90 minutes. Cells were then incubated for 30 minutes with secondary antibodies plus DAPI at 200 ng/mL in 50% blocking buffer in PBS. Total protein was stained with 1 μM Alexa488-NHS or Alexa647-NHS in 50 mM carbonate buffer at a pH of 9.2 for 15 minutes.

#### mRNA poly(A) fluorescence in situ hybridisation

Assays were performed similarly to previous descriptions.^4^ All steps were followed by three PBS washes (cell fixation and permea-bilisation) or 2X saline sodium citrate (SSC) buffer (FISH steps; Thermo Fisher Invitrogen AM9763). Cells were fixed in 4% paraformaldehyde (EMS Emgrid 15710) for 15 minutes, then permeabilised in 70% ethanol at 4 ºC for 4–6 hours. Total protein was stained with 1 μM Alexa488-NHS in a 50 mM carbonate buffer at a pH of 9.2 for 15 minutes.

Cells were washed twice with FISH wash buffer containing 10% formamide (Thermo Fisher Invitrogen AM9342) in 2X SSC. Cells were hybridised overnight at 37 ºC with 100 nM ATTO647N-labelled poly-dT (Integrated DNA Technologies) in a hybridisation buffer containing 10% formamide by volume (Thermo Fisher Invitrogen AM9342), 2 mM ribonucleoside vanadyl complexes (New England Biolabs S1402S), 100 μg/mL yeast transfer RNAs (Thermo Fisher Invitrogen 15401011), 200 μg/mL BSA (Thermo Fisher Invitrogen AM2616), and 100 mg/mL dextran sulphate (Merck Sigma Aldrich D8906-50G) in 2X SSC. The next day, two one-hour washes at 37 ºC were performed in FISH wash buffer, the second containing DAPI at 200 ng/mL. A single room temperature wash in FISH wash buffer was performed, followed by washing three times in 2X SSC alone, which cells were left in for imaging.

#### 5-ethynyl uridine visualisation via click chemistry

Assays were performed as previously described.^4^ All steps were followed by three PBS washes. Cells were fixed in 4% paraformaldehyde (EMS Emgrid 15710) for 15 minutes, then permeabilised in 0.25% Triton X100 (Sigma Aldrich 93443) for 10 minutes. Cells were changed into Tris-buffered saline (125 mM sodium chloride, 50 mM Tris pH 8 Thermo Fisher Invitrogen AM9856) via 3x wash. A 1.5x click reaction mixture was made in TBS containing 150 mM sodium ascorbate (Sigma Aldrich A7631), 3 mM copper sulphate (Chem-Supply Australia CA068) and 7.5 μM Alexa647 azide (Thermo Fisher Invitrogen A10277). 30 μL of 1.5x click reaction was added onto 15 μL residual TBS and incubated for 30 minutes at room temperature. Total protein was stained with 1 μM Alexa488-NHS in 50 mM carbonate buffer at a pH of 9.2 for 15 minutes, with DAPI added for 5 minutes at 200 ng/mL in PBS.

#### Fixed cell imaging

For experiments on HCT116 cells with siRNA knockdown of ARMC5 (Figure S2A), and in combination with compound treatment (Figure 3B), imaging was performed on a Perkin Elmer Operetta CLS, with 40x/NA1.1 water immersion objective and LED light source.

For all other immunofluorescence, EU click, and poly(A) FISH experiments, on HCT116 mCherry-RPB1 and HEK293 parental and *ARMC5* knock-out cells, imaging was performed on a Nikon Ti2 microscope equipped with a Yokogawa CSU-W1 spinning disk, with 40x/NA0.95 Plan Apo λ air objective, and dual Hamamatsu ORCA-Fusion C14440-20UP cameras. 20 z-planes at 1 μm intervals were acquired. DAPI DNA stain was acquired with a 405 nm laser and 450/82 nm filter. Alexa488+ conjugated secondary antibodies were acquired with a 488 nm laser and 525/50 nm filter, and mCherry-RPB1 was acquired with a 561 nm laser and 617/73 nm filter. Where applicable, Alexa488-NHS or Alexa647-NHS cell stains were acquired with the appropriate green (525/50 nm) or far-red (685/ 40 nm) filter.

#### Fluorescence recovery after photobleaching (FRAP)

3 days after plating cells in 8-well chamber-slides, regular media was exchanged for 360 μL imaging media, McCoy’s 5A phenol red-free (Cytiva SH30270.01) + 10% FBS (Moregate Biotech) + 1% penicillin and streptomycin (Sigma-Aldrich P0781) via a 2x wash on HCT116 mCherry-RPB1 cells. Where included, triptolide, triptolide plus CB-5083, and vehicle-only were made up in imaging media at 10x concentrations and added into wells 60 minutes before commencing imaging of that well, 40 μL onto 360 μL. Final concentrations were 1 μM triptolide, ± 10 μM CB-5083, in a consistent 0.044% DMSO vehicle in all wells in experiments with compound treatment. All FRAP traces were collected on a Zeiss LSM900 point-scanning confocal with Plan-Apochromat 63x oil immersion objective, NA 1.40, at 37_C and 5% CO_2_, in a window of 60 – 90 minutes after compound treatment where relevant. For each cell, an initial image of the whole nucleus was collected, before two circular regions were imaged with a diameter of 1.8 μM (18 pixels) and an area of 2.45 μM (255 pixels). Both regions were imaged within a 1 s frame for 120 s. After a 10 frame baseline, one region was bleached with 100% laser power for approximately 5 s. Control traces were collected under identical conditions from cells fixed in 4% PFA for 15 minutes. See quantification and statistical analysis for a description of image analysis.

Loss of fluorescence intensity during acquisition in the unbleached control region was due to fluorophore depletion during photo-bleaching step, and not due to photobleaching during acquisition, as this did not occur in fixed cells (Figure S3D), nor did it occur when photobleaching was not performed (Figure S3A).

Following triptolide treatment, mCherry-RPB1 fluorescence intensity was dramatically reduced. In order to perform the FRAP experiment in the triptolide-alone condition, some selection bias was introduced in the experimenter choosing cells with observable residual fluorescence. Following INTS8 knockdown, either alone or in combination with ARMC5 knockdown, there was a notable effect on cell health. Unhealthy appearing cells, which were poorly attached and rounded, were not assayed.

For initial experiments comparing siRNA knockdown of ARMC5 to scrambled control (Figure 2H), five experiments were performed with ten cells collected per condition, per experiment. For experiments comparing the effect of triptolide following ARMC5 knockdown (Figures 5B and 5C), three experiments were performed with 7 – 10 cells collected per condition, per experiment. For experiments comparing ARMC5 knockdown to INTS8 knockdown and in combination (Figures 6E, 6F, S7B and S7C), five experiments were performed with 10 cells collected per condition, per experiment.

### Quantification And Statistical Analysis

#### Quantitative image processing

For initial experiments on HCT116 with siRNA knockdown of ARMC5 (Figure S2A), and in combination with compound treatment (Figure 3B), analysis was performed within Operetta CLS Harmony software (Perkin Elmer). Illumination bias was corrected, z-stacks maximum-intensity projected and nuclei segmented using the DAPI channel. Mean fluorescence intensity was then taken per cell, which was analysed and plotted as below.

All other image processing except FRAP was done using a custom pipeline written in python, which progresses from raw images through to extraction of single-cell measurements. These were then analysed, summarised and plotted using RStudio, making use of the tidyverse packages.^93^ Z-stacks were maximum-intensity projected and corrected for illumination biases across the field-of-view, as previously described.^94^ Nuclei were segmented in 2D from the DAPI signal using a manually trained Cellpose 2.0 model (based on the ‘nuclei’ model). Separate models were used for HEK293 cells and HCT116 cells. Cell segmentation was done for poly(A) FISH experiments, and was achieved by watershed-based segmentation of the poly(A) FISH signal using nuclei as seeds, making use of the mahotas python package.^91^ Mean fluorescence intensity and nuclear morphology measurements were calculated using the regionprops function from the scikit-image python package.^90^ Nuclei touching image borders were excluded.

To combine data from replicate experiments performed on different days, quantitative measurements were normalised by dividing all background-subtracted data on the plate by the mean of control wells (e.g., either ‘Vehicle/HEK293’ or ‘Scrambled siRNA’).

#### Protein half-life measurement using bleach-chase

The bleach-chase method was performed similarly to the original method,^53^ which involves tracking the dynamics of a fraction of ‘invisible’ mCherry-RPB1,$\tilde{P}(t)$, that is created during a bleach pulse and is degraded thereafter. Since it is not visible, its levels are inferred at each time point from the difference between total protein levels in unbleached cells, *P(t)*, and the visible protein levels in the bleached cells, *P*_*v*_*(t)*, according to,

$$\tilde{P}(t)=P(t)-P_{v}(t)$$

If removal of $\tilde{P}$ is constant in time:

$$\tilde{P}(t)=\tilde{P}(0)\left(1-e^{-\alpha_{tot\;}t}\right),$$

where α_*tot*_ is the total removal rate of mCherry-RPB1. α_*tot*_ is most conveniently estimated by fitting the equation:

(Equation 1) $$\ln\left(P(t)-P_{v}(t)\right)=\ln\left(P(0)-P_{v}(0)\right)-\alpha_{tot}t$$

to experimental data. In growing cells, protein concentration decreases via both degradation and dilution, so the total removal rate measured, *α*_*tot*_, is the sum of the degradation rate, *α*_*deg*_, and the dilution rate, *α*_*dil*_.

After acquiring images as described in method details, Z-stacks were maximum-intensity projected and nuclei were segmented in 2D from the mCherry-RPB1 signal using a manually trained Cellpose 2.0 model^95^ (based on the ‘nuclei’ model). Mean fluorescence intensity in each nucleus at each timepoint was calculated using the regionprops function from scikit image.^90^ After removing all cells touching the image borders, 6000-17000 cells at each timepoint were quantified. Background fluorescence intensity was estimated from a region outside the cells and was subtracted from intensity values. The amount of invisible mCherry-RPB1, $\tilde{P}(t)$, generated during the bleach pulse was estimated by averaging across cells from bleached and unbleached regions (separately) in each well, and subtracting the mean intensity of mCherry-RPB1 in bleached regions *P*_*v*_(*t*) from that of cells in unbleached regions, *P*(*t)*. The mean loss of fluorescence intensity over the 9h chase (acquisition photobleaching) was estimated from unbleached cells, to be 6 ± 1%, in both siRNA treatments.

The mean nuclear intensity of ‘invisible’ mCherry-POLR2A, $\tilde{P}(t)$, was well described by an exponential decay model over the 9h recovery, as indicated by a linear fit of Equation 1 to the data (Figure S2I). This indicates constant turnover kinetics during the 9h chase. a_*tot*_ was estimated by fitting Equation 1 to the data using a linear mixed effects models (Satterthwaite’s degrees-of-freedom method) in the lme4 package^96^ in R. Each well was treated as a random effect on both slope and intercept. A chi-squared test indicated a significant difference in slopes (*α*_*tot*_) between scrambled siRNA and ARMC5 siRNA treated cells (P<10^-6^). Estimates of mean together with 95% confidence intervals for *α*_*tot*_ in each condition were obtained from the fitted model using the emmeans package.^97^ The rate of protein dilution due to cell growth, *α*_*dil*_, was estimated by fitting an exponential growth model to the number of cells in each field-of-view:

(Equation 2) $$N(t)=N(0)e^{\alpha_{dil}t}$$

Again, we used linear mixed effects models in the lme4 package to estimate α_*dil*_ (after log-transforming Equation 2). Similarly to the procedure above, each well was treated as a random effect for both slope and intercept. A chi-squared test indicated that ARMC5 siRNA transfection did not have a significant effect on cell growth rate (P=0.53), but we noticed a small effect of bleaching on cell growth: α_*dil*_ = 0:040 ± 0:001 compared to *α*_*dil*_ = 0:043 ± 0:001 for unbleached (P=0.001; equivalent to a 1.3h lengthening of the cell cycle). Because recovery is calculated from the bleached cells, with unbleached cells serving as a (typically constant) reference, e11 Molecular Cell *84*, 4808–4823.e1–e13, December 19, 2024 we used *α*_*dil*_ = 0:040 ± 0:001, from the bleached cells for protein half-life calculations. Errors indicate the 95% confidence interval for the mean.

This fitted value of *α*_*dil*_ is equivalent to a doubling time of *T*_*D*_ = *ln* 2=a_*dil*_ = 17:2± 0:6 h which agrees well with doubling times obtained previously for HCT116 cells, of 17.1 h^98^ or 17.4 h.^99^

Mean rates of protein turnover due to active degradation in each condition were calculated as:

$$\alpha_{deg}=\alpha_{tot}-\alpha_{dil\;},$$

with uncertainty in α_*deg*_ estimated as,

$$\frac{\delta \alpha_{deg\;}}{\alpha_{deg\;}}={\left({\left(\frac{\delta \alpha_{tot\;}}{\alpha_{tot\;}}\right)}^{2}+{\left(\frac{\delta \alpha_{dil\;}}{\alpha_{dil\;}}\right)}^{2}\right)}^{1/2},$$

where δ*α*_*tot*_ and δ*α*_*dil*_ are the uncertainties in turnover and dilution rates, respectively. a_*deg*_ was finally converted to a protein half-life using *T*_1=2_ = *ln* 2=a_*deg*_.

Protein concentration *P* = *α*_*syn*_=*α*_*tot*_ where *α*_*syn*_ is the protein synthesis rate. Leaving the synthesis rate unchanged, and changing the removal rate α_*tot*_/*α*_*tot*_^*´*^, the new protein concentration will be *P*^*´*^ = *α*_*syn*_=α_*tot*_^*´*^. Therefore *P*^*´*^=*P* = *α*_*tot*_=α_*tot*_^*´*^, so the new protein concentration is modified by a factor equal to the ratio of the two removal rates. In the case of cells transfected with ARMC5 siRNA, *α*_*tot* (*Scrambled*)_=*α*_*tot* (*ARMC*5)_ = 1.51 (95% confidence interval: 1.38-1.64), which gives an expected change in protein abundance if there is no synthesis rate change (Figure 2D).

#### FRAP analysis

Representative images of pre- and post-FRAP trace collection in fixed cells are shown in Figure S3B. Raw fluorescence intensity values of FRAP traces from all cells for scrambled control and ARMC5 knockdown conditions, in both live and fixed conditions, are shown in Figure S3C. Each trace was individually normalised as a percentage of the pre-bleach baseline values, as shown in Figure S3D. FRAP traces collected from fixed cells did not show substantial recovery, reflecting almost completely immobilised mCherry-RPB1 (Figure S3D). Further rescaling of FRAP traces was performed, with 0% being defined by the post-bleaching intensity in fixed cells, and 100% being defined by the intensity of the respective unbleached control region at each timepoint (Figure S3E).

Normalised, rescaled FRAP traces were fit in Prism 9 (Graphpad Software) with a two-phase association model, fitting either each cell individually, or the mean of each experimental day (Figure S3F). The initial value was constrained to the 0% value defined by fixed cells, and the plateau was constrained to the 100% value defined by the unbleached control region.

When kinetic rates of the two components were allowed to vary across experimental day and between conditions in comparing control and ARMC5-depleted cells, estimated half-lives of the two components did not substantially vary (Figure S2G). Additionally, when fitting the mean data, fast and slow half-lives as a shared parameter between scrambled and ARMC5 depletion conditions was the preferred model over a model where these parameters varied between conditions (Extra sum-of-squares F test, F (DFn, DFd) = 1.203 (2, 994), p = 0.3). As a result the kinetic rates of the two components were shared across experimental conditions for both percell and per-experiment fitting. The increase in the fraction of freely diffusing RPB1 following ARMC5 knockdown shown via curve fitting (Figure 2I) is also observable by simply quantifying the rapid initial recovery of fluorescence 15 s after bleaching (Figure S3G).

To calculate fluorescence intensity-adjusted Pol II fractions, estimates of the bound percentage of Pol II were multiplied by the normalised baseline fluorescence intensity of each condition within each experiment relative to the corresponding control. Mean with range across experiments are shown.

### Computational analysis of genome-wide experiments

#### dxChIP-seq alignment and processing

dxChIP-seq reads were trimmed and quality-filtered with Trim Galore (https://github.com/FelixKrueger/TrimGalore), using a quality threshold of 30. Trimmed reads were aligned to the hg38 genome using Bowtie^2,77,78^; default parameters, then PCR duplicates were marked and removed with Picard (http://broadinstitute.github.io/picard). The correlation between replicates was checked using deepTools multiBamSummary,^80^ then replicates were merged.^79^ Merged BAM files were converted into RPKM-normalised bigwigs using deepTools bamCoverage.

#### dxChIP-seq metagene profiles and quantification

Ensembl-annotated genes (GRCh38.102) were stratified by gene length (where appropriate) and split into bins. TSSs and TTSs were defined using a ±500bp window (>1kb genes), while gene bodies were defined by excluding 2kb segments at the start and end of each gene (>5kb genes). Coverage was computed using bedtools,^81^ normalising to the average signal 5kb upstream of each gene (metagene analysis) or the average signal outside of genes and Pol II peaks (meta TSS/TTS/gene body quantification). All down-stream data processing and visualisation was performed in R, using dplyr^82^ and ggplot2.^83^

#### Pol II pausing index analysis

Pausing indices were calculated as the dxChIP-seq read density at the TSS ( − 30 bp to +300 bp) divided by the read density at the gene body (+700 to TTS) for each protein-coding gene longer than 1.5kb in length.^71^ Genes were only considered for analysis if they had a TSS read coverage of at least 5-fold over background in at least one condition. All data processing and visualisation was performed in R, using dplyr and ggplot2.

#### TTchem-seq alignment and processing

TT_chem_-seq reads were trimmed and quality-filtered with Trim Galore, using a quality threshold of 30. Trimmed reads were aligned to the hg38 and *Saccharomyces cerevisiae* (sacCer3) genomes using STAR aligner^84^ with basic two-pass mapping. PCR duplicates were marked and removed using Picard, and the correlation between replicates was checked using deepTools multiBamSummary. Replicates were merged, and resulting BAM files were split by strand.^79^ Bigwig files were created using deepTools bamCoverage, normalising to the number of spike-in reads.

#### TTchem-seq metagene profiles

Metagene analysis followed the same protocol as dxChIP-seq, with the inclusion of an extra step to detect and cap extreme outliers beyond the interquartile range multiplied by 100. Spike-in normalised bigwigs were used for mapping, and metagenes were plotted without further background normalisation.

#### TTchem-seq quantification and differential expression analysis

For each replicate, read counts per gene (including introns) were determined using the htseq-count tool.^85^ Pairwise analyses were performed with DESeq2,^86^ incorporating spike-in normalisation for quantitative comparisons across samples. Low-count genes were captured by prefiltering for genes with average normalised counts of at least 10, then performing the differential expression analysis with independent filtering switched off. All comparisons were made against the WT siCtrl condition, identifying significantly regulated genes based on an adjusted *p*-value threshold of 0.05 (Benjamini and Hochberg method) and a minimum fold-change of 2. All downstream data processing and visualisation was performed in R, using dplyr and ggplot2.

#### Analysis of gene length and baseline expression

Significantly upregulated and downregulated genes were compared to the set of prefiltered genes used for differential expression analysis (‘all expressed genes’) across several metrics. The length of each gene was determined using Ensembl annotations (GRCh38.102), and baseline expression was defined as the average number of normalised read counts across all biological replicates in untreated WT cells. To remove redundancy between categories, genes individually regulated by either ARMC5 or INTS8 were subtracted from the combined set of ARMC5 + INTS8 genes (where appropriate). Venn diagrams were constructed using eulerr,^87^ and boxplots were visualised using ggplot2.

#### Gene ontology analysis

Gene ontology analysis was conducted with the clusterProfiler package.^88,89^ *P*-values underwent correction by the Benjamini-Hochberg method, and significant GO terms were identified based on a q-value threshold of 0.05.

#### Analysis of promoter types

Core promoter elements were determined using classifications from the Eukaryotic Promoter Database.^100,101^ The prevalence of differentially expressed genes across these annotations was visualised using GraphPad Prism.

## Supplementary Material

Document S1. Figures S1-S8.

Document S2. Article plus supplemental information.

## Highlights

- CRL3^ARMC5^ ubiquitylates RNA Pol II to regulate its levels off-DNA and at gene starts
- CRL3^ARMC5^ targets perturbed, incompetent RNA Pol II complexes
- Integrator phosphatase compensates for the loss of ARMC5
- ARMC5 and Integrator prevent the release of incompetent RNA Pol II into late elongation

## In brief

Cacioppo, Gillis, Shlamovitz, Zeller, et al. show that CRL3^ARMC5^ ubiquitylates RNA Pol II at early stages of transcription as part of a major homeostatic RNA Pol II turnover mechanism. CRL3^ARMC5^ and the Integrator phosphatase act as complementary pathways, monitoring the quantity and quality of RNA Pol II complexes before they are licensed into elongation.
