# Supplementary material for: CRL3ARMC5 ubiquitin ligase and Integrator phosphatase form parallel mechanisms to control early stages of RNA Pol II transcription
Source: Mol Cell. Author manuscript; Available in PMC 2025 Feb 25. (PMC7617427; doi:10.1016/j.molcel.2024.11.024)
Supplement: Document S1. Figures S1-S8. [file EMS203404-supplement-Document_S1__Figures_S1_S8_.pdf]

Molecular Cell, Volume *84*

Supplemental information

CRL3^{ARMC5} ubiquitin ligase and Integrator phosphatase form parallel mechanisms to control early stages of RNA Pol II transcription

Roberta Cacioppo, Alexander Gillis, Iván Shlamovitz, Andrew Zeller, Daniela Castiblanco, Alastair Crisp, Benjamin Haworth, Angela Arabiotorre, Pegah Abyaneh, Yu Bao, Julian E. Sale, Scott Berry, and Ana Tufegdžić Vidaković

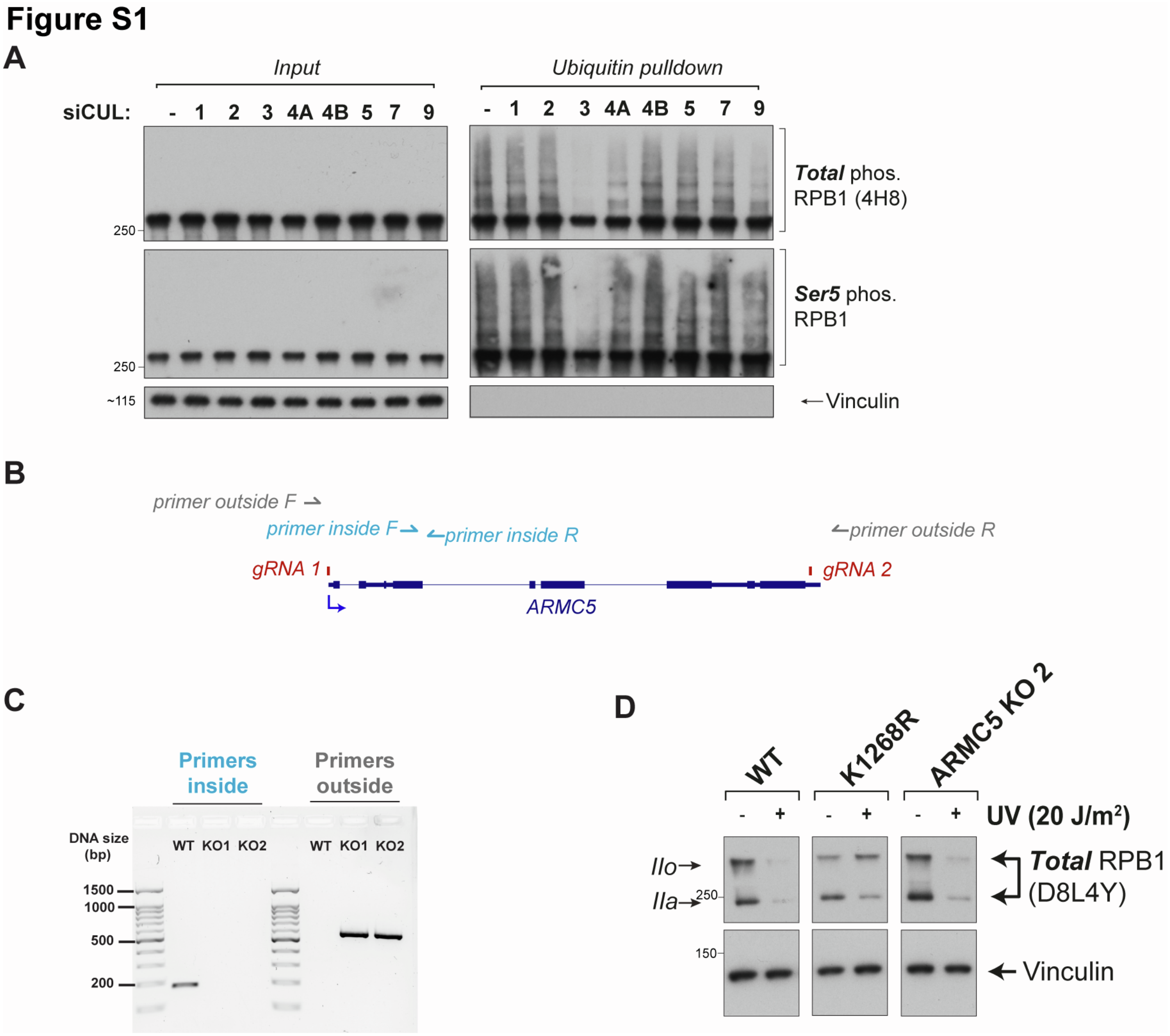

Figure S1. Distinct forms of ubiquitylated Pol II in the transcription cycle. Related to Figure 1.
(A) As in Figure 1E but without CB-5083 treatment.
(B) Position of gRNAs used to excise the *ARMC5* locus using CRISPR-Cas9, and the primers used for screening.
(C) Validation of *ARMC5* gene deletion via PCR using primers outlined in (B).
(D) Western blot analysing stability of total RPB1, before and 8 h after UV (20 J/m^2), in WT, RPB1 K1268R and *ARMC5* KO cells.

Figure S2

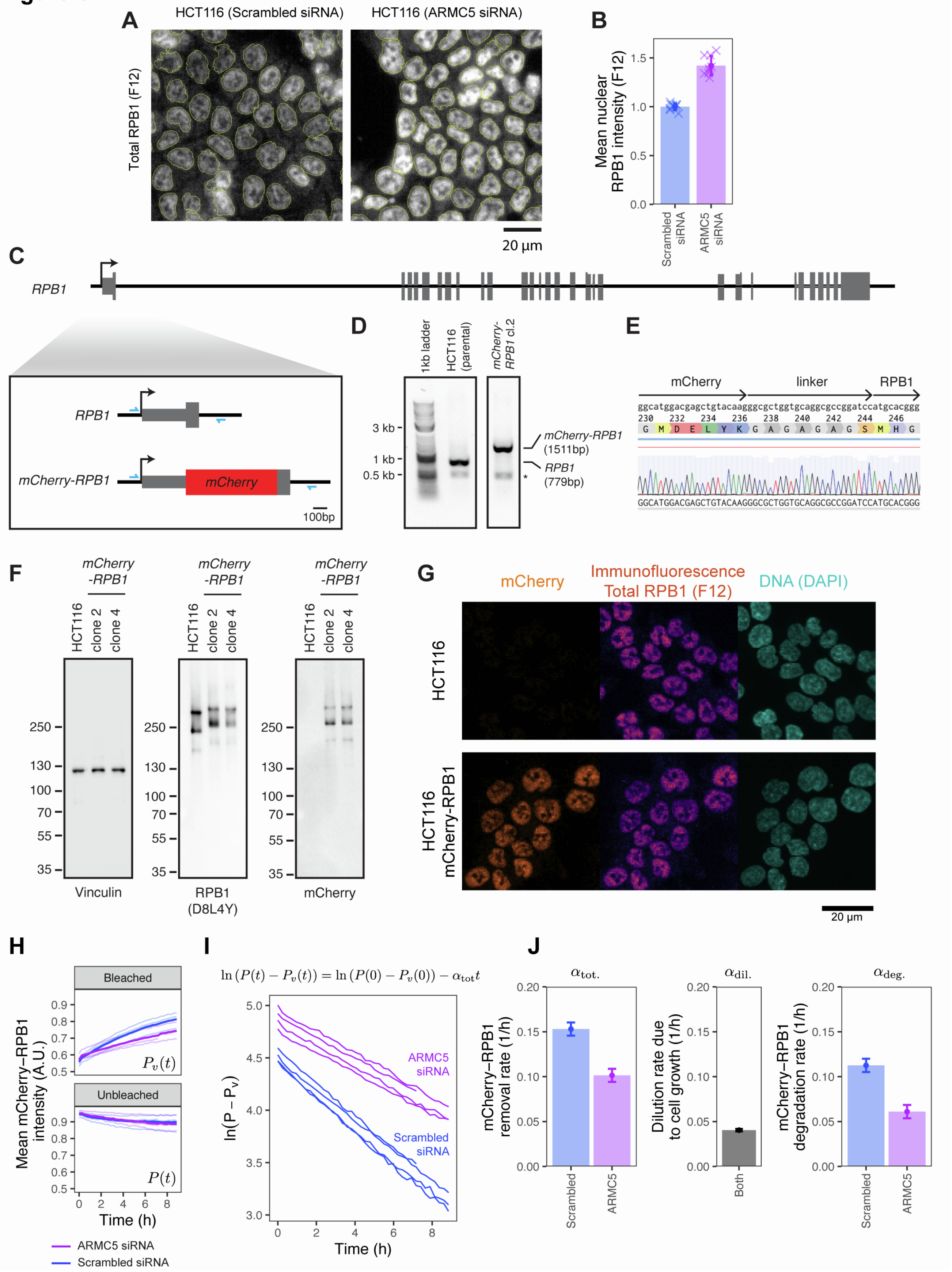

Figure S2. Generation of mCherry-RPB1 cells. Related to Figure 2.

(A) Total RPB1 (F12) immunofluorescence in HCT116 cells, transfected with siRNA targeting *ARMC5* or negative control scrambled siRNA.

(B) Quantification of (A). Values normalised to the mean intensity of cells transfected with scrambled siRNA in each experiment. Crosses show means from individual replicate wells with bar height representing the overall mean of nine wells measured across three experiments. Error bars show +/- SD.

(C) Genomic structure of the *RPB1* gene, showing insertion site for mCherry fusion protein. Positions of primers used for PCR genotyping shown in blue.

(D) PCR genotyping homozygous *mCherry-RPB1* (clone 2) by amplifying across insertion site using primers depicted in A. * indicates non-specific band.

(E) Sanger sequencing across the *mCherry-RPB1* fusion from genomic DNA extracted from *mCherry-RPB1* clone 2 cells.

(F) Western blots of HCT116 *mCherry-RPB1* and parental HCT116 cells, with antibodies as shown.

(G) Example images of mCherry-RPB1 and total Pol II immunofluorescence (F12) in the same cells for *mCherry-RPB1* cells and parental HCT116 cells.

(H) Quantification of mean nuclear mCherry-RPB1 intensity during bleach-chase time-lapse experiments used to measure RPB1 protein stability. Upper panel shows recovery of bleached regions ($P_V(t)$). Lower panels show intensity of unbleached regions imaged in the same well ($P(t)$) (6% loss of intensity in lower panel is due to acquisition photobleaching). Intensity values normalised to pre-bleach intensity. Mean across 6000-17000 cells for each experiment. Four experiments shown as partially transparent lines, with the overall mean across experiments at each time-point shown as a darker solid line.

(I) Total mCherry-RPB1 removal rate, $\alpha_{tot.}$ measured by determining the slope from the plot, as depicted in the equation above. Each line represents one of the four experiments, with the plotted value representing the mean across 6000-17000 cells. Unlike in H, data are not normalised to pre-bleach intensity before plotting.

(J) Calculated values for $\alpha_{tot.}$,$\alpha_{dil.}$ and $\alpha_{deg.}$. Error bars represent 95% confidence intervals for the mean, derived from linear mixed effects models (STAR Methods). Cellular growth rates represented by $\alpha_{dil.}$ did not depend on whether cells were transfected with *ARMC5* siRNA or negative control scrambled siRNA.

Figure S3

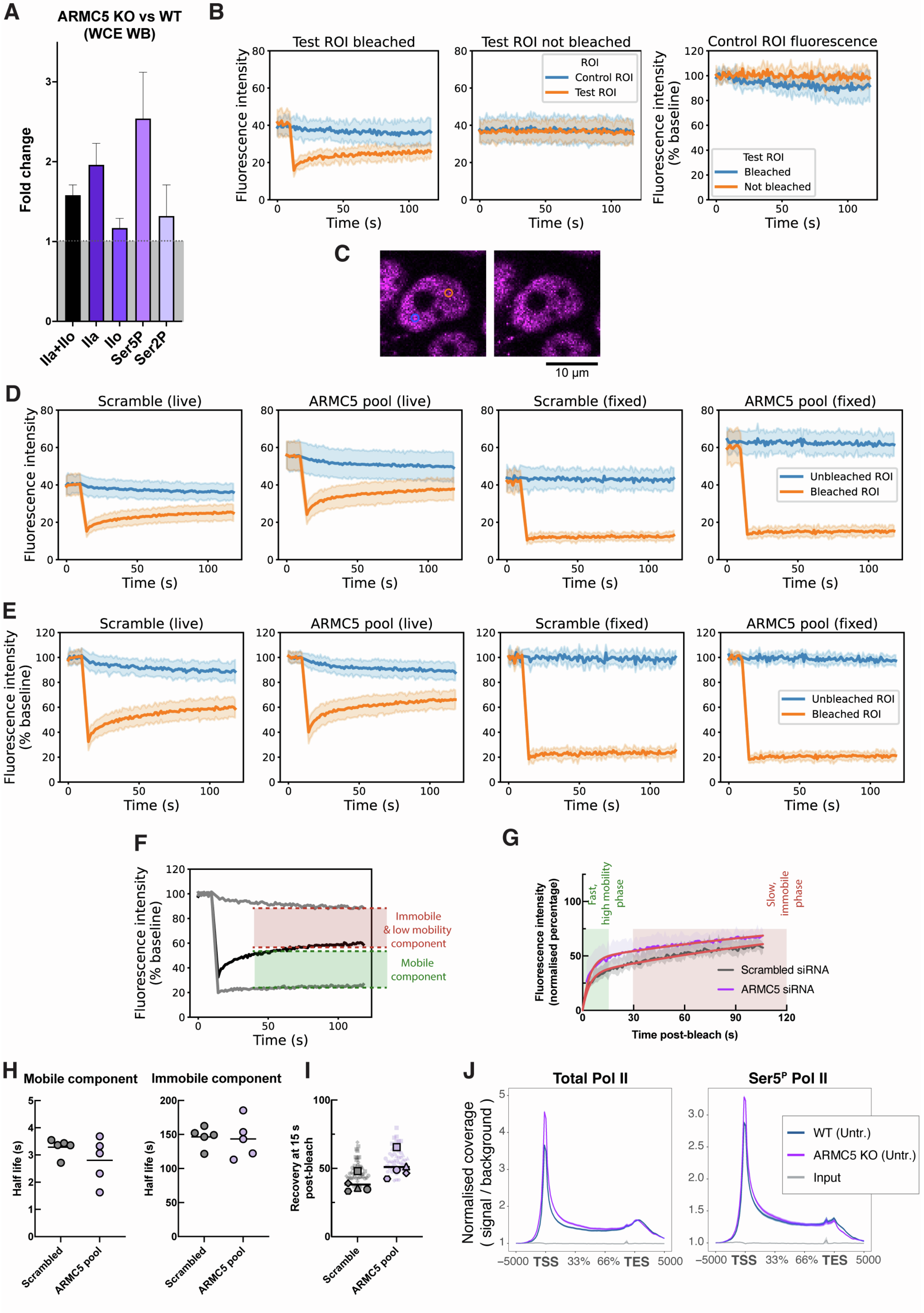
A
ARMC5 KO vs WT (WCE WB)
Fold change
IIa+IIo
IIa
IIo
Ser5P
Ser2P
B
Test ROI bleached
Test ROI not bleached
Control ROI fluorescence
Fluorescence intensity
Fluorescence intensity (% baseline)
Time (s)
ROI
Control ROI
Test ROI
Test ROI
Bleached
Not bleached
C
10 µm
D
Scramble (live)
ARMC5 pool (live)
Scramble (fixed)
ARMC5 pool (fixed)
Unbleached ROI
Bleached ROI
E
F
Immobile & low mobility component
Mobile component
G
Fast, high mobility phase
Slow, immobile phase
Fluorescence intensity (normalised percentage)
Time post-bleach (s)
Scrambled siRNA
ARMC5 siRNA
H
Mobile component
Immobile component
Half life (s)
Scrambled
ARMC5 pool
I
Recovery at 15 s post-bleach
Scramble
J
Total Pol II
Ser5P Pol II
Normalised coverage (signal / background)
WT (Untr.)
ARMC5 KO (Untr.)
Input
TSS
TES
33%
66%

Figure S3. ARMC5 controls the levels of free and promoter-proximal Pol II. Related to Figure 2.

(A) Quantification of IIa, IIo, Ser5^{P} and Ser2^{P} from whole cell lysate western blots of RPB1. Three biological replicates were analysed using ImageJ, standard deviation is plotted.

(B) FRAP control traces where the test ROI was either bleached or not bleached. Plots show mean and standard deviation of total 10 cells per condition collected in two separate experiments.

(C) Example image from fixed cells showing mCherry-RPB1 signal pre- and post-bleach. Black circles denote ROIs.

(D) Raw fluorescence intensity FRAP traces collected in mCherry-RPB1 cells transfected with either pooled ARMC5 or scrambled control siRNAs, in either live or fixed conditions. Plots show mean and standard deviation of total 50 cells (live) or total 20 cells (fixed), collected in 5 (live) or 2 (fixed) separate experiments.

(E) Normalised FRAP traces, same data as in (C) shown as percentage of the pre-bleach baseline.

(F) Mean FRAP trace from 50 live scrambled siRNA control cells, with data collected from 20 fixed cells and intensity of the unbleached control ROI overlaid in grey, representing bounds of the fluorescence recovery.

(G) Mean post-bleach FRAP traces for *ARMC5* knockdown or scrambled siRNA control conditions, normalised using data from fixed cells and the unbleached ROI, with a two-component exponential association fit overlaid in red. Plot shows mean with range across 5 experiments, with 10 cells collected per experiment.

(H) Summary plots from fitting of individual experiments for *ARMC5* knockdown or scrambled siRNA control conditions where the half life of the two components was permitted to vary. Points represent one of five experiments, bars represent the mean.

(I) Normalised intensity values after 15 s post-bleach recovery, from curves normalised as in F. Large points represent data per experiment (5 experiments), small points represent data per cell (50 cells total), bars represent mean.

(J) Metagene profiles showing the distribution of Pol II occupancy across gene units in dxChIP-seq. Untreated condition is plotted, from a larger experiment encompassing treatment and shown later in Figures 4 and S5.

Figure S4

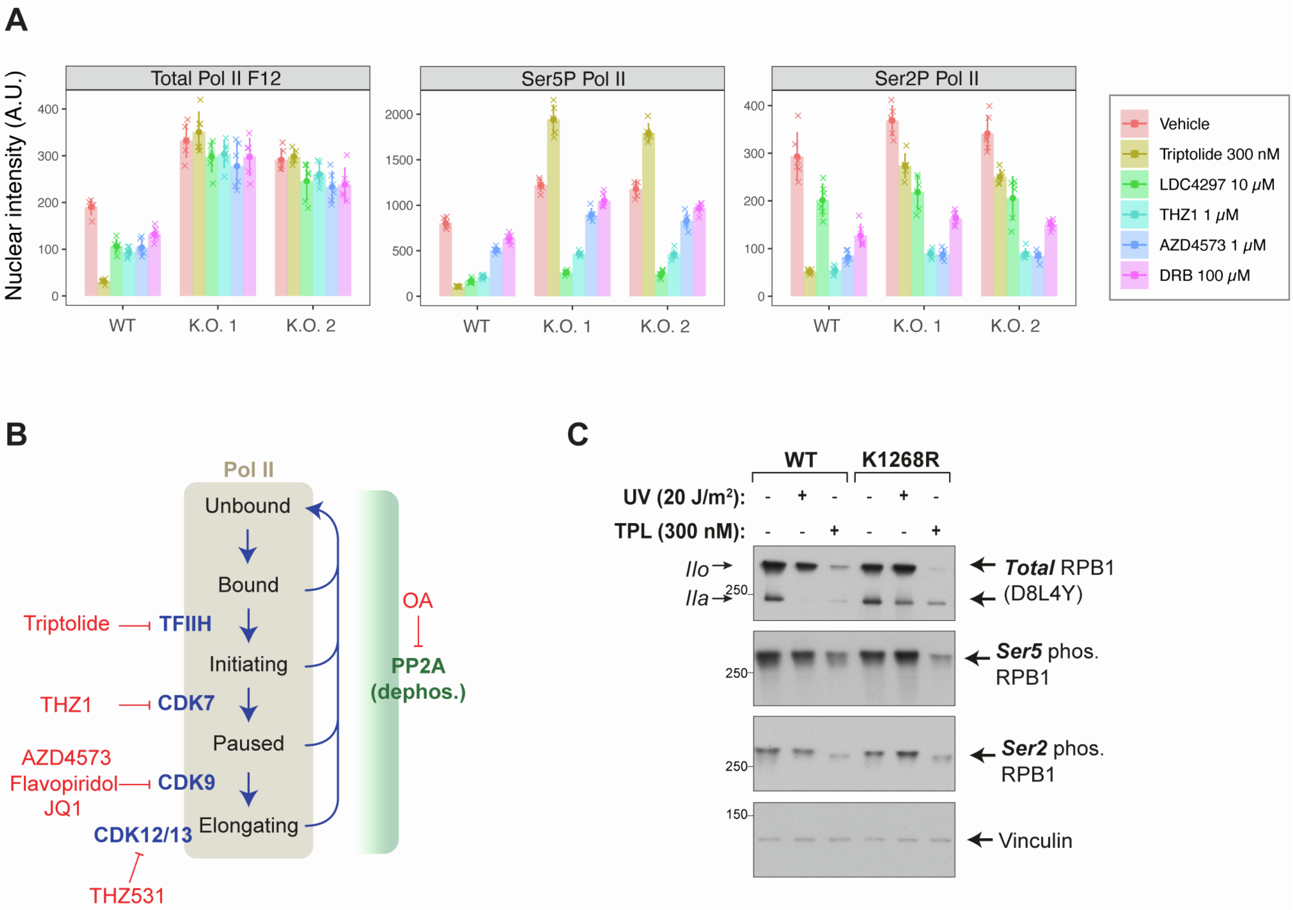

Figure S4. ARMC5 targets perturbed early transcription complexes. Related to Figure 3.

(A) Mean nuclear intensity of total, Ser5^P, and Ser2^P RPB1, detected via immunofluorescence, in either *ARMC5* knock-out or WT HEK293 cells treated with the indicated compound or 0.4% DMSO vehicle for 4 hours. Mean +/- SD of three experiments conducted in duplicate shown, with each replicate displayed as a cross.

(B) Schematic of the transcription cycle indicating steps targeted by the inhibitors used in ubiquitin-pulldown experiments.

(C) Western blot analysing the stability of total RPB1, upon UV (3 h, 20 J/m^2) and triptolide (TPL, 300 nM, 2 h) treatments.

Figure S5

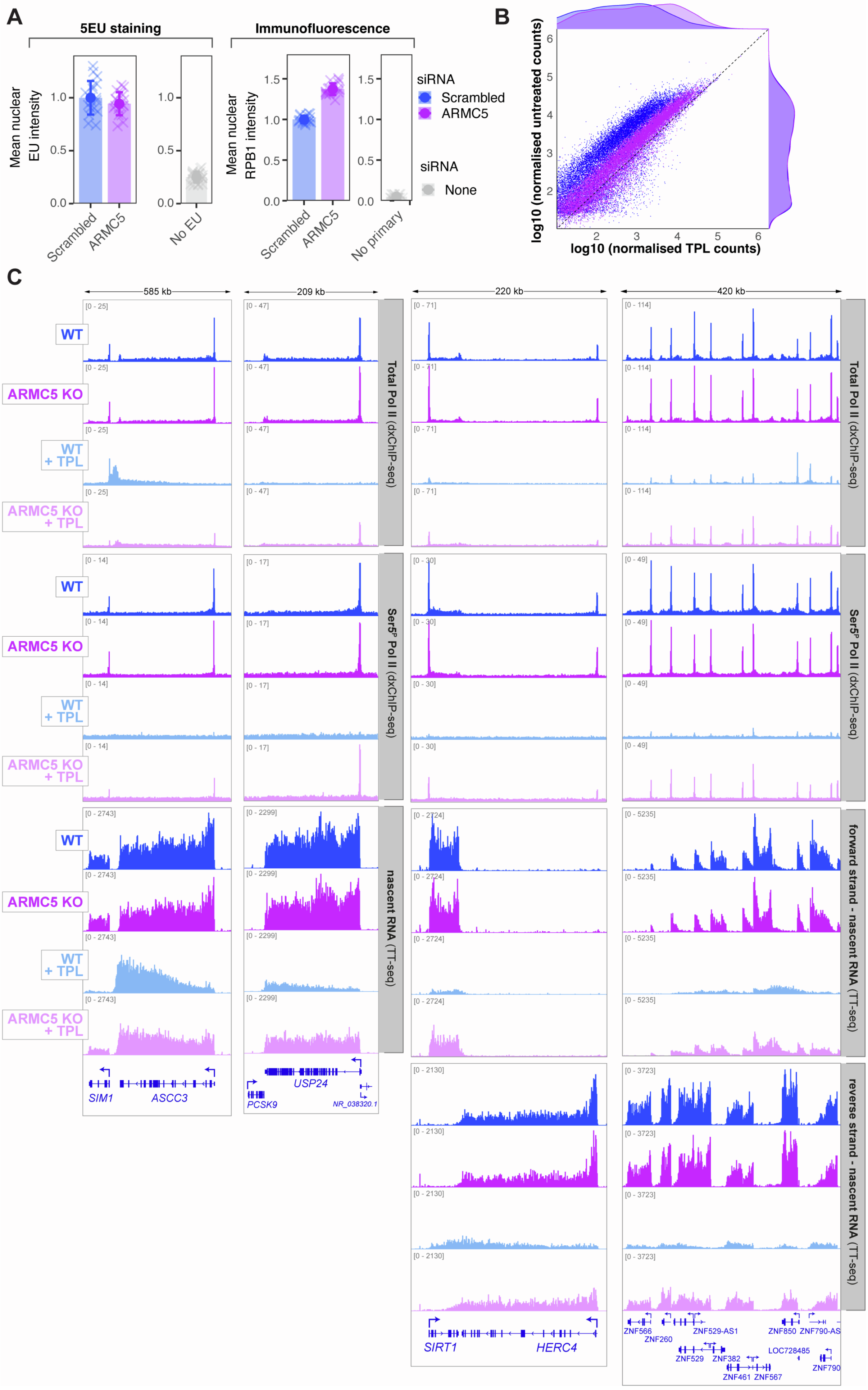
A
5EU staining
Immunofluorescence
Mean nuclear EU intensity
Mean nuclear RPB1 intensity
Scrambled
ARMC5
No EU
No primary
siRNA
Scrambled
ARMC5
siRNA
None
B
log10 (normalised untreated counts)
log10 (normalised TPL counts)
C
585 kb
209 kb
220 kb
420 kb
WT
ARMC5 KO
WT + TPL
ARMC5 KO + TPL
Total Pol II (dxChIP-seq)
Ser5P Pol II (dxChIP-seq)
nascent RNA (TT-seq)
forward strand - nascent RNA (TT-seq)
reverse strand - nascent RNA (TT-seq)
SIM1
ASCC3
USP24
PCSK9
NR_038320.1
SIRT1
HERC4
ZNF566
ZNF260
ZNF529-AS1
ZNF850
ZNF790-AS
ZNF529
ZNF382
LOC728485
ZNF790
ZNF461
ZNF567

Figure S5. ARMC5 loss confers partial resistance to XBP inhibition by triptolide. Related to Figure 4.

(A) Left: Mean nuclear 5-EU intensity for cells pulse-labelled for 30 min. Values normalised to the mean intensity of HCT116 transfected with Scrambled siRNA. Crosses show means from individual replicate wells (500-2000 cells/well) with bar height representing the overall mean of 5-6 wells measured across three experiments. Error bars show +/- SD. Right: immunofluorescence of total RPB1 in same wells in which 5EU intensity was measured.

(B) Scatterplot and marginal density plots comparing the number of TT_{chem}-seq reads at expressed genes (>10 normalised reads) in wild-type (blue) and *ARMC5* KO (purple) cells; x-axis - log10 spike-in normalised counts (triptolide treated cells); y-axis - log10 spike-in normalised counts (untreated cells).

(C) Individual gene examples from dxChIP-seq (top, middle) and TT_{chem}-seq (bottom) experiments, panels on the right show nascent RNA derived from both DNA strands as regions displayed contain dense clusters of genes in both directions.

Figure S6

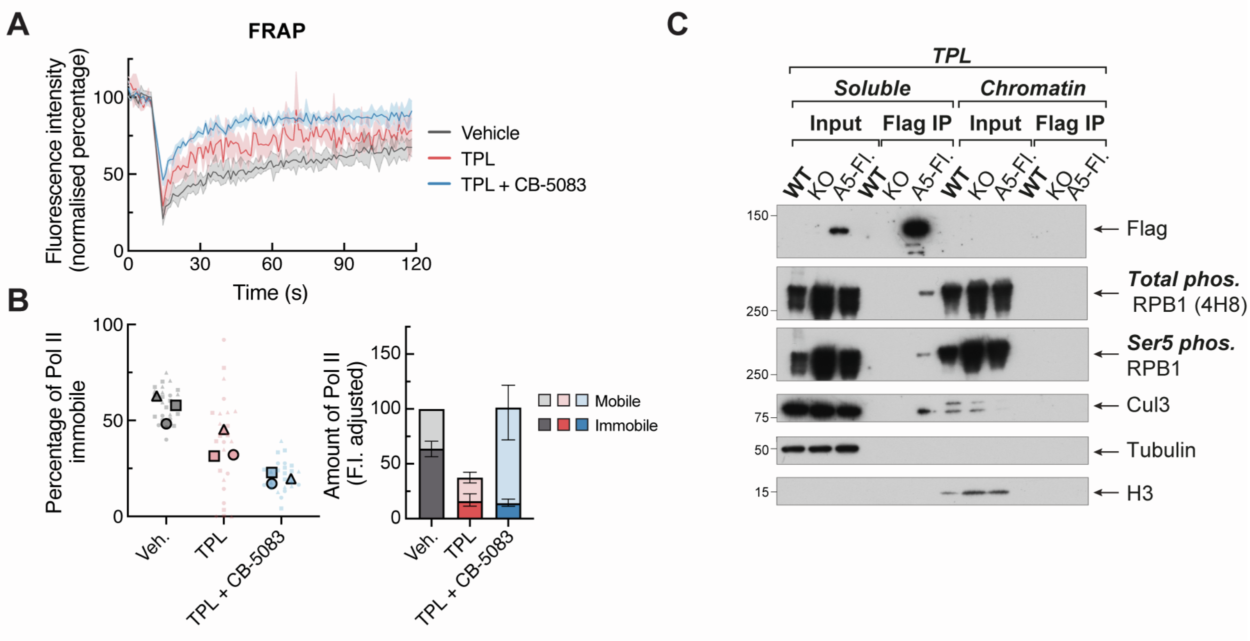

Figure S6. Evicted, phosphorylated Pol II accumulates off-chromatin in the absence of ARMC5. Related to Figure 5.

(A and B) As in Figures 5B and 5C, respectively, for cells treated with 1 µM triptolide (TPL), with or without 10 µM CB-5083 p97i.

(C) Chromatin fractionation followed by Flag IP and Western blot in WT, *ARMC5* KO, or *ARMC5* KO cells expressing exogenous ARMC5-Flag, treated with triptolide (300 nM) for 1h. No crosslinker was used in this experiment.

Figure S7

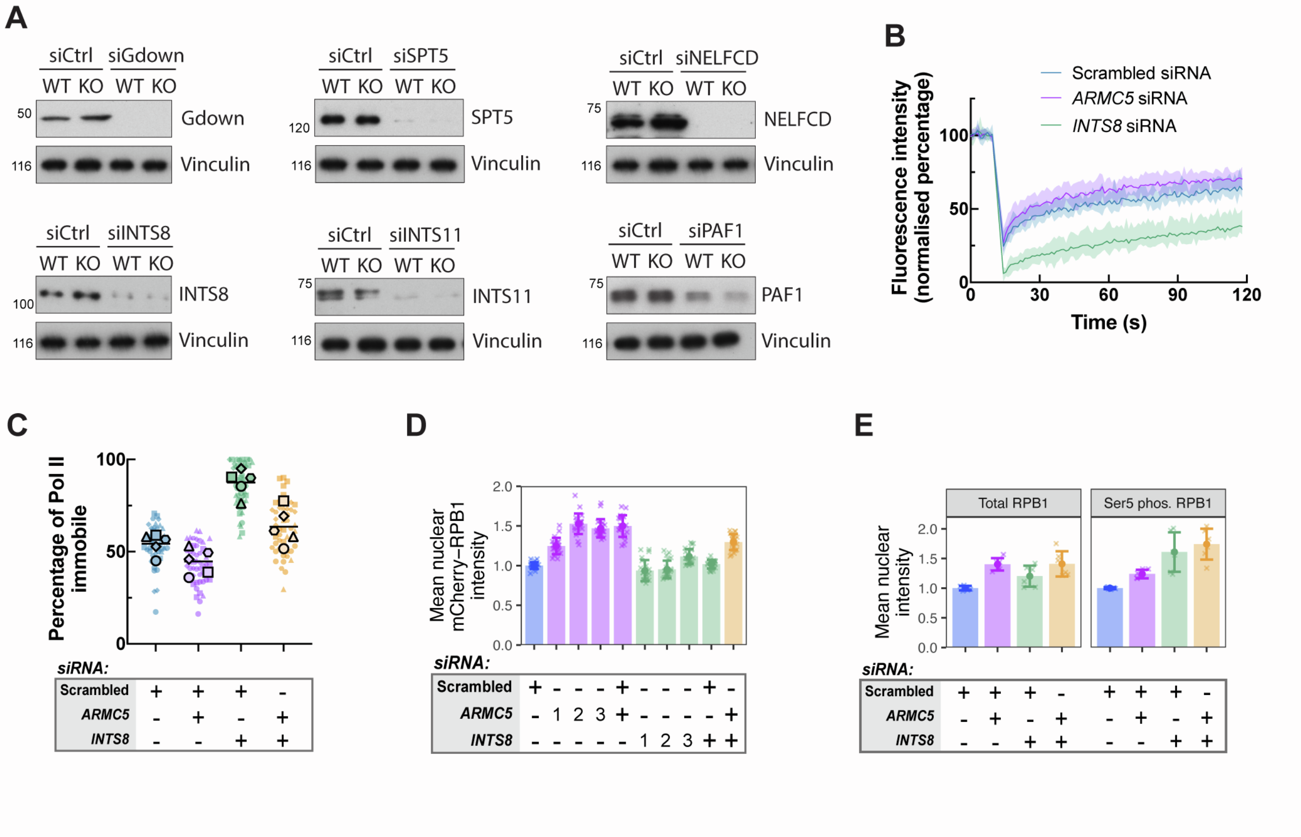

Figure S7. Integrator phosphatase module compensates for the loss of ARMC5. Related to Figure 6.

(A) Western blot analysis of siRNA-mediated knock-down efficiency for indicated factors, in wild-type (WT) and *ARMC5* KO cells. (KO).

(B) Kinetic FRAP traces of mCherry-RPB1 cells following ARMC5 knockdown and INTS8 knockdown (scrambled siRNA was used as a control). Plots show mean with range of five experiments with 10 cells collected per experiment.

(C) Fraction of RPB1 in the immobile component, following ARMC5 knockdown and INTS8 knockdown, alone or in combination, or scrambled siRNA control. Small points and large points represent fitting per cell (50 cells total) and per experiment (5 experiments) respectively, bars represent mean.

(D) mCherry intensity in fixed HCT116 *mCherry-RPB1* cells. Crosses show means from individual replicate wells (500-2500 cells/well), with bar height representing the overall mean of 16 wells measured across two experiments. Error bars show +/- standard deviation. Numbers shown below represent for assays of the individual siRNAs that comprise the ARMC5 and INTS8 siRNA ‘pools’.

(E) Immunofluorescence measurements from HCT116 mCherry-RPB1 cells. Crosses show means from individual replicate wells (500-2500 cells/well), with bar height representing the overall mean of four wells measured across two experiments. Error bars show standard deviation. RPB1 Total, F12 antibody; RPB1 Ser5 phos., 3E8 antibody.

Figure S8

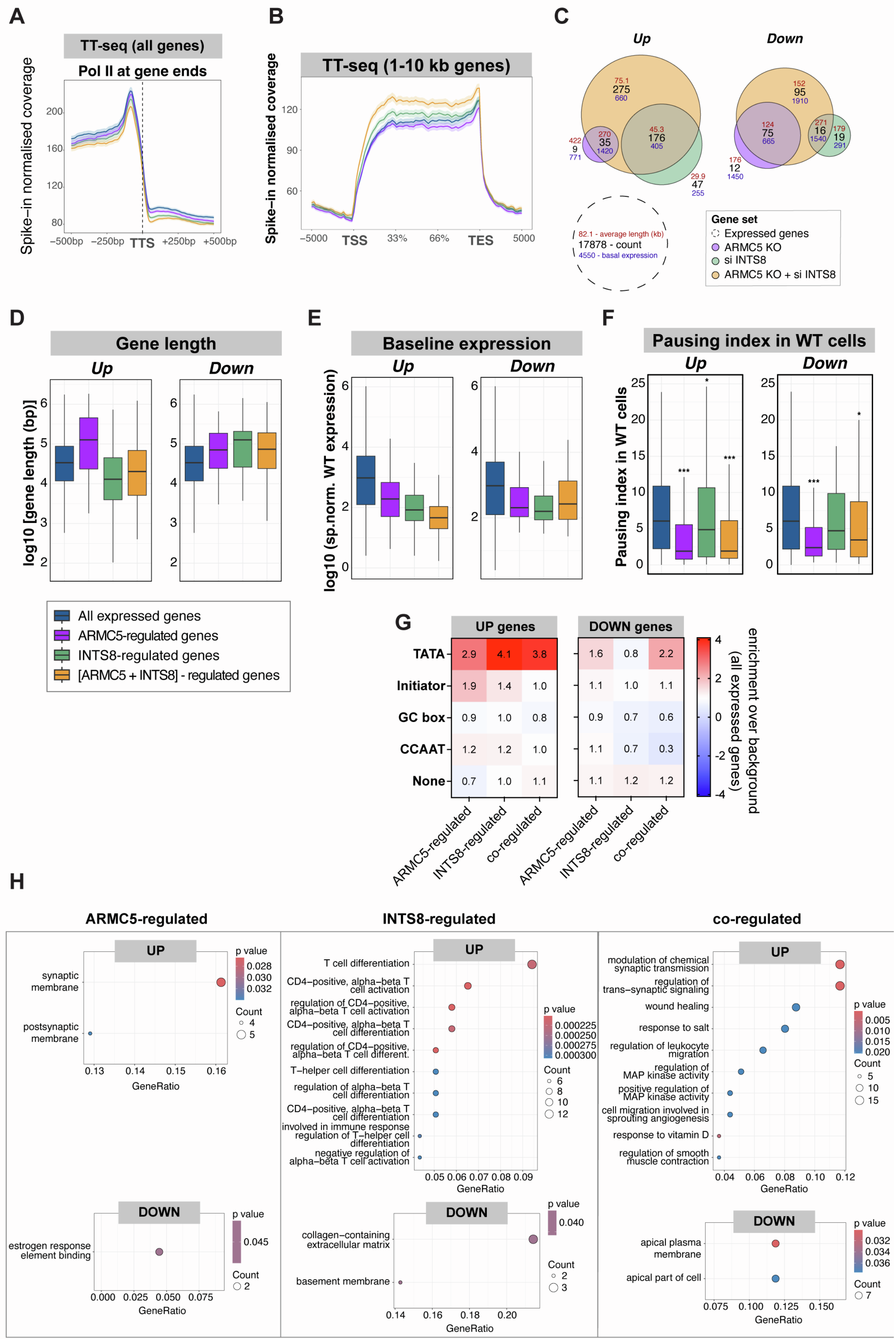

A
TT-seq (all genes)
Pol II at gene ends
Spike–in normalised coverage
-500bp -250bp TTS +250bp +500bp
B
TT-seq (1-10 kb genes)
Spike–in normalised coverage
-5000 TSS 33% 66% TES 5000
C
Up
Down
82.1 - average length (kb)
17878 - count
4550 - basal expression
Gene set
Expressed genes
ARMC5 KO
si INTS8
ARMC5 KO + si INTS8
D
Gene length
Up
Down
log10 [gene length (bp)]
All expressed genes
ARMC5-regulated genes
INTS8-regulated genes
[ARMC5 + INTS8] - regulated genes
E
Baseline expression
Up
Down
log10 (sp.norm. WT expression)
F
Pausing index in WT cells
Up
Down
Pausing index in WT cells
G
UP genes
DOWN genes
TATA
Initiator
GC box
CCAAT
None
ARMC5-regulated
INTS8-regulated
co-regulated
enrichment over background (all expressed genes)
H
ARMC5-regulated
INTS8-regulated
co-regulated
UP
DOWN
synaptic membrane
postsynaptic membrane
estrogen response element binding
T cell differentiation
CD4−positive, alpha−beta T cell activation
regulation of CD4−positive, alpha−beta T cell activation
CD4−positive, alpha−beta T cell differentiation
regulation of CD4−positive, alpha−beta T cell different.
T−helper cell differentiation
regulation of alpha−beta T cell differentiation
CD4−positive, alpha−beta T cell differentiation involved in immune response
regulation of T−helper cell differentiation
negative regulation of alpha−beta T cell activation
collagen−containing extracellular matrix
basement membrane
modulation of chemical synaptic transmission
regulation of trans−synaptic signaling
wound healing
response to salt
regulation of leukocyte migration
regulation of MAP kinase activity
positive regulation of MAP kinase activity
cell migration involved in sprouting angiogenesis
response to vitamin D
regulation of smooth muscle contraction
apical plasma membrane
apical part of cell
p value
Count
GeneRatio

Figure S8. ARMC5 and INTS8 regulate the quantity and quality of early transcription complexes. Related to Figure 7.

(A) Absolute scale metagene TT_{chem}-seq profiles showing nascent RNA distribution at gene ends, in WT and *ARMC5* KO cells, transfected with siINTS8 or control siRNA.

(B) Relative scale metagene TT_{chem}-seq profiles showing nascent RNA distribution across genes shorter than 10 kb, in WT and *ARMC5* KO cells, transfected with siINTS8 or control siRNA.

(C) Venn diagrams showing gene count, average length and baseline expression of significantly upregulated and downregulated genes.

(D) Boxplots showing the distribution of lengths of differentially expressed genes.

(E) Boxplots showing the distribution of baseline expression levels (in WT cells) of differentially expressed genes.

(F) Boxplots showing the distribution of the pausing index (in WT cells) of differentially expressed genes.

(G) Heatmap showing the representation of different core promoter elements in differentially expressed gene sets. Fold change in frequency of each core promoter element versus background (all expressed genes) is plotted.

(H) Gene ontology analysis showing processes enriched amongst differentially expressed gene sets. The top ten hits ($q<0.05$), sorted and colour coded by BH-corrected p value, are displayed for each condition.
